# The *B. subtilis* Rok protein is an atypical H-NS-like protein irresponsive to physico-chemical cues

**DOI:** 10.1093/nar/gkac1064

**Published:** 2022-11-21

**Authors:** Amanda M Erkelens, Liang Qin, Bert van Erp, Andrés Miguel-Arribas, David Abia, Helena G J Keek, Dorijn Markus, Marc K M Cajili, Samuel Schwab, Wilfried J J Meijer, Remus T Dame

**Affiliations:** Leiden Institute of Chemistry, Leiden University, Einsteinweg 55, 2333CC Leiden, The Netherlands; Centre for Microbial Cell Biology, Leiden University, Leiden, The Netherlands; Centre for Interdisciplinary Genome Research, Leiden University, Leiden, The Netherlands; Leiden Institute of Chemistry, Leiden University, Einsteinweg 55, 2333CC Leiden, The Netherlands; Centre for Microbial Cell Biology, Leiden University, Leiden, The Netherlands; Centre for Interdisciplinary Genome Research, Leiden University, Leiden, The Netherlands; Leiden Institute of Chemistry, Leiden University, Einsteinweg 55, 2333CC Leiden, The Netherlands; Centre for Microbial Cell Biology, Leiden University, Leiden, The Netherlands; Centre for Interdisciplinary Genome Research, Leiden University, Leiden, The Netherlands; Centro de Biología Molecular “Severo Ochoa” (CSIC-UAM), C. Nicolás Cabrera 1, Universidad Autónoma, Canto Blanco, 28049 Madrid, Spain; Bioinformatics Facility, Centro de Biología Molecular “Severo Ochoa” (CSIC-UAM), C. Nicolás Cabrera 1, Universidad Autónoma de Madrid, Canto Blanco, 28049 Madrid, Spain; Leiden Institute of Chemistry, Leiden University, Einsteinweg 55, 2333CC Leiden, The Netherlands; Leiden Institute of Chemistry, Leiden University, Einsteinweg 55, 2333CC Leiden, The Netherlands; Leiden Institute of Chemistry, Leiden University, Einsteinweg 55, 2333CC Leiden, The Netherlands; Centre for Microbial Cell Biology, Leiden University, Leiden, The Netherlands; Centre for Interdisciplinary Genome Research, Leiden University, Leiden, The Netherlands; Leiden Institute of Chemistry, Leiden University, Einsteinweg 55, 2333CC Leiden, The Netherlands; Centre for Microbial Cell Biology, Leiden University, Leiden, The Netherlands; Centre for Interdisciplinary Genome Research, Leiden University, Leiden, The Netherlands; Centro de Biología Molecular “Severo Ochoa” (CSIC-UAM), C. Nicolás Cabrera 1, Universidad Autónoma, Canto Blanco, 28049 Madrid, Spain; Leiden Institute of Chemistry, Leiden University, Einsteinweg 55, 2333CC Leiden, The Netherlands; Centre for Microbial Cell Biology, Leiden University, Leiden, The Netherlands; Centre for Interdisciplinary Genome Research, Leiden University, Leiden, The Netherlands

## Abstract

Nucleoid-associated proteins (NAPs) play a central role in chromosome organization and environment-responsive transcription regulation. The *Bacillus subtilis*-encoded NAP Rok binds preferentially AT-rich regions of the genome, which often contain genes of foreign origin that are silenced by Rok binding. Additionally, Rok plays a role in chromosome architecture by binding in genomic clusters and promoting chromosomal loop formation. Based on this, Rok was proposed to be a functional homolog of *E. coli* H-NS. However, it is largely unclear how Rok binds DNA, how it represses transcription and whether Rok mediates environment-responsive gene regulation. Here, we investigated Rok's DNA binding properties and the effects of physico-chemical conditions thereon. We demonstrate that Rok is a DNA bridging protein similar to prototypical H-NS-like proteins. However, unlike these proteins, the DNA bridging ability of Rok is not affected by changes in physico-chemical conditions. The DNA binding properties of the Rok interaction partner sRok are affected by salt concentration. This suggests that in a minority of *Bacillus* strains Rok activity can be modulated by sRok, and thus respond indirectly to environmental stimuli. Despite several functional similarities, the absence of a direct response to physico-chemical changes establishes Rok as disparate member of the H-NS family.

## INTRODUCTION

The bacterial chromosome, like the chromosomes of eukaryotic cells, is highly organized and compactly folded. At the same time, housekeeping genes need to be accessible for the transcription machinery while other (sets of) genes must become available for transcription upon changing environmental conditions. This requires a flexible and dynamic organization of the bacterial chromosome in response to environmental cues (1–3). Many factors contribute to the organization and compaction of the nucleoid, including DNA supercoiling, macromolecular crowding and nucleoid-associated proteins (NAPs) (4–6). The histone-like nucleoid structuring protein (H-NS), one of the main NAPs in *Escherichia coli*, plays important roles in both chromosome organization and gene regulation (7,8). H-NS can non-specifically bind DNA across the genome, but has a preference for AT-rich DNA. DNA acquired via horizontal gene transfer (HGT) is often AT-rich and is therefore recognized as xenogeneic DNA by H-NS (9,10). Although genes acquired via HGT are key to the evolution of bacteria by conferring new genetic traits, inappropriate transcription of acquired genes can lead to loss of competitive fitness. H-NS family proteins, which include H-NS of *E. coli*, MvaT of *Pseudomonas* sp. and Lsr2 of *Mycobacteria*, function as silencers of xenogeneic genes, until repression is relieved by particular environmental signals (11–13).

Rok of *Bacillus subtilis*, which was originally identified as the repressor of competence regulator *comK* (14), plays a role in the repression of genes for cell surface and extracellular functions (15). Rok has been proposed to be a functional homolog of H-NS, based on the observation that it binds preferentially to AT-rich regions of the chromosome acquired via horizontal gene transfer (16). Rok contributes to silencing of the genes within such regions (16), including those encoding antimicrobial compounds (17). Rok also decreases chromosomal transformation (14), possibly by altering nucleoid architecture (18). This behavior classifies Rok as a xenogeneic silencer like H-NS, MvaT and Lsr2. Rok primarily silences expression by interfering with the initial steps of transcription (19). In contrast, H-NS also binds intragenic regions to repress spurious transcription. Such repression by Rok is less needed in *B. subtilis* due to enhanced specificity of its RNA polymerase (19). Lastly, Rok was found to be associated with a large subset of chromosomal domain boundaries in *B. subtilis* (20), suggesting that it contributes to chromosome organization similarly as was proposed previously for H-NS (21,22). Recently, it was demonstrated that Rok indeed establishes long-range chromosomal loops in *B. subtilis* (23). This is suggestive of Rok acting as a DNA bridging protein. However, the molecular basis underlying the precise roles of Rok in transcriptional regulation and chromosome organization and their possible interplay remains unknown. In addition, it is unknown whether gene silencing by Rok can be modulated by changes in physico-chemical growth conditions such as temperature, pH and salt. A distinct group of *rok* genes has been identified on several *Bacillus* plasmids belonging to the pLS20 family and on some *Bacillus* chromosomes (24,25). Because these *rok* genes are smaller, we refer to them as small Rok (sRok) (8). The most outstanding difference between the two types of Rok is that sRok lacks a part of Rok's linker domain. sRok can associate with the host chromosome and it can replace Rok in the competence pathway (24). Therefore, we hypothesized that sRok has similar DNA binding features as Rok.

In this study, we used a combined *in vitro* (single-molecule) and *in vivo* approach to study the DNA binding properties of Rok, sRok and the artificial Rok Δ75–96 variant. We found that Rok and sRok are both DNA bridging proteins, but that they respond differently to changes in environmental conditions. We show that the DNA binding properties of Rok are only very mildly modulated by physico-chemical changes, while the DNA bridging activity of sRok is osmo-sensitive. We also demonstrate that Rok and sRok can form heterodimers and show that these interactions alter DNA bridging properties, which in turn, most probably, affect the regulatory role of Rok and sRok *in vivo*. Therefore, interactions with sRok and possibly other interactions with proteins might be key to the regulation of Rok-mediated gene repression.

## MATERIALS AND METHODS

### Cloning and mutagenesis

Oligonucleotides used for cloning and mutagenesis procedures are listed in Supplementary Table S1. The Rok coding sequence from *B. subtilis* 168 (Ref seq: NC_000964.31493787–1494362, Uniprot O34857, NCBI protein database: NP_389307.1) was cloned into pET30b using Gibson Assembly (26) resulting in plasmid pRD231. This plasmid was used as template to create the expression vector for Rok Δ75–96 (pRD415) using Gibson Assembly, where amino acids 75–96 were deleted from the sequence. pRD231 was also used to create plasmid pRD461 containing Rok with a C-terminal His-tag (Rok 6xHis) using Gibson assembly. The coding sequence for sRok was created and codon optimized with GeneArt (Thermo Fisher Scientific) (Uniprot: E9RJ31 NCBI protein database: BAJ76946.1) and cloned into pET30b using Gibson assembly creating plasmid pRD411. The plasmids for *in vivo* complementation use the pUC19 vector as backbone. All inserts into pUC19 were cloned into the vector using Gibson Assembly. pRD408 was created by taking the *E. coli* MG1655 *hns* promoter including the upstream regulatory region up to position -150 (genomic location 1292358–1292508) followed by the H-NS coding sequence (genomic location 129509–129923). The Rok, sRok and Rok Δ75–96 plasmids (pRD424, pRD410 and pRD412) were created by replacing the H-NS coding sequence of pRD408 with the Rok, sRok or Rok Δ75–96 sequence from pRD231, pRD411 or pRD415 respectively. In a previous publication, pRD424 was named pRok (19). The sequence of all constructs was verified by DNA Sanger sequencing (BaseClear). All plasmids were deposited at Addgene and their information and identification numbers are summarized in Supplementary Table S2.

### DNA substrates

Tethered particle motion and bridging assay experiments were performed using an AT-rich (32% GC) 685 bp DNA substrate described earlier (27,28) unless otherwise stated. The DNA substrate was generated by PCR using Thermo Scientific^®^ Phusion^®^ High-Fidelity DNA Polymerase and the products were purified using the GenElute PCR Clean-up kit (Sigma-Aldrich). As single-stranded DNA substrates for the bridging assay, parts with comparable GC-content (around 32%) of the 685 bp DNA substrate were ordered as oligonucleotides (Sigma-Aldrich) and turned into double-stranded DNA using PCR and complementary oligonucleotides (Supplementary Table S3). The poly(A) single-stranded DNA with an average length between 250 and 500 bp was ordered from Sigma-Aldrich and turned into double-stranded DNA using PCR and poly(T) oligonucleotides. For use in the DNA bridging assay (see below), DNA was ^32^P-labeled (29). For microscale thermophoresis, complementary oligonucleotides of 78 bp were designed (Supplementary Table S4) and the top strand was 5’ labelled with Cy5. The oligonucleotides were mixed 1:1 to a final concentration of 40 μM, heated to 95°C and slowly cooled down to room temperature for annealing. For Atomic Force Microscopy, pUC19 plasmid was incubated with nicking endonuclease Nb.BsrDI (New England Biolabs) for one hour at 65°C followed by heat inactivation at 80°C for 20 minutes and cooling to room temperature. The nicked plasmid was then purified by phenol chloroform extraction and the buffer was exchanged with HPLC water (Sigma-Aldrich) through overnight dialysis at RT using a Slide-A-Lyzer cassette with a 3.5 kDa cut-off (Thermo Scientific).

### Protein purification

*E. coli* BL21(DE3) pLysS cells, transformed with pRD231, pRD411 or pRD415 were grown at 37°C, 250 rpm until an OD_600_ (optical density at 600 nm) of 0.6. Expression was induced with 1 mM IPTG and cell growth was continued at 16°C, 180 rpm overnight. Cells were pelleted at 6354 × g, 4°C and resuspended in 20 mM Tris–HCl pH 8.0, 130 mM NaCl, 10% glycerol with 100 μM PMSF and 20.5 μg/ml DNase I. The cells were lysed using a French press and the lysate was centrifuged with an ultracentrifuge (Beckman Coulter) for 30 min at 100 736 × g. The supernatant was filtered with a 0.22 μm Millex-GP Syringe Filter and loaded on a HiTrap Heparin HP 1 ml affinity column (GE Healthcare). The Rok protein was eluted using an NaCl gradient from 130 mM to 1.5 M. The eluted fractions were checked for the presence of Rok with SDS-PAGE and the relevant fractions were pooled, concentrated with an Amicon 10 kDa cut-off filter and buffer exchanged with a PD10 column (GE Healthcare) to a buffer with 130 mM NaCl. Next, the protein was loaded on and eluted from a HiTrap SP HP 1 mL column (GE Healthcare) using a NaCl gradient from 130 mM to 1.5 M. The fractions were again checked with SDS-PAGE and concentrated to 500 μl with an Amicon 10 kDa cut-off filter. The protein was then loaded on a GE Superdex 200 10/300 Increase GL column pre-equilibrated with storage buffer (20 mM Tris–HCl pH 8.0, 300 mM KCl, 10% glycerol). Rok Δ75–96 and sRok were purified according to the same general protocol with minor modifications. For both proteins, the pH of the buffers (except the storage buffer) was changed to 7.5 to increase affinity for the columns. For sRok, 3 μM benzamidine was added to the lysis buffer to prevent cleavage and a P11 column using a gradient from 100 mM to 1.5 M NH_4_Cl was used first. The eluate containing sRok was dialysed overnight against 20 mM Tris–HCl pH 8.0, 130 mM NaCl, 10% glycerol and the purification continued with a Heparin column as described. Cells that expressed Rok with a C-terminal his-tag (Rok-6xHis, pRD461) were lysed in 20 mM Tris–HCl pH 8, 300 mM NaCl, 10 mM imidazole, 3.5% glycerol. Rok-6xHis was purified using a 5 ml HisTrap HP column (GE Healthcare) with a gradient from 10 mM to 1 M imidazole, followed by gel filtration as described above. Protein concentrations were determined using a Pierce™ BCA Protein Assay Kit (Thermo Scientific) or a Qubit™ Protein assay kit (Invitrogen). Purity (>95%) and identity of the proteins was verified with SDS-PAGE and mass spectrometry.

### Tethered particle motion

The DNA used for Tethered Particle Motion (TPM) experiments was an AT-rich (32% GC) 685 bp DNA substrate (30,31). Measurements were performed as previously described (30,32) with minor modifications. Briefly, the flow cell was washed with 100 μL experimental buffer (10 mM Tris–HCl pH 8.0, 10 mM EDTA, 5% glycerol, 50 mM KCl) to remove excess beads and 100 μl protein diluted in experimental buffer was flowed in and incubated for 5 min. Next, the flow cell was washed with protein solution one more time, sealed with nail polish and incubated for 5 min. After incubation, the flow cell was directly transferred to the holder and incubated for 5 more minutes in the instrument to stabilize the temperature at 25°C for the measurement. For each flow cell, more than 200 beads were measured and measurements for each concentration were performed at least in duplicate.

Data analysis including the calculation of occupancy was done as described previously (30). The occupancy (*θ*) is the fraction of DNA bound by Rok (Equation 1) and can be calculated from the area under the peaks of the fitted Gaussian distributions.

(1) $$\begin{equation*}\theta = \frac{{{A_{peak,\;bound}}}}{{{A_{peak,\;bound}} + {A_{peak,\;unbound}}}}\;\end{equation*}$$

In which, *θ* is the occupancy and *A* is the area under the peaks of the fitted Gaussian distribution. Fitting the occupancies as a function of the concentration of Rok using the Hill-binding model (Equation 2) yields the apparent binding affinity *K_D_* and Hill coefficient (*n*).

(2) $$\begin{equation*}\theta \left( c \right) = \frac{1}{{{{\left( {\frac{{{K_D}}}{{\left[ c \right]}}} \right)}^n} + 1}}\;\end{equation*}$$

In which, *θ(c)* is the occupancy, *K_D_* is the apparent binding affinity, [*c*] is the ligand concentration, and *n* is the Hill coefficient.

### Bridging assay

The DNA used for the bridging assay is the same as that used for TPM and was ^32^P-labeled (29). The DNA bridging assay was performed as described previously (27,33) with minor modifications. Streptavidin-coated Magnetic M-280 Dynabeads (Invitrogen) were resuspended in buffer (20 mM Tris–HCl pH 8.0, 2 mM EDTA, 2 M NaCl, 2 mg/ml BSA (ac), 0.04% Tween 20) containing 100 fmol biotinylated 32% GC DNA (685 bp) and incubated at 1000 rpm for 20 min at 25°C in an Eppendorf Thermomixer with an Eppendorf Smartblock™ 1.5 ml. The beads with associated DNA were washed twice before resuspension in buffer (10 mM Tris–HCl, pH 8.0, 5% v/v glycerol, 1 mM spermidine, 0.02% Tween20, 1 mg/ml acetylated BSA). Radioactive ^32^P-labeled DNA and unlabeled DNA were combined to maintain a constant (2 fmol/μl) concentration and a radioactive signal around 8000 cpm, and then added to each sample. Next, protein was added to initiate formation of bridged protein-DNA complexes. Salt concentration (KCl or MgCl_2_), protein concentration, temperature and pH were individually varied in line with the experiments. For pH 6 and 6.5 10 mM MES (2-morpholinoethanesulfonic acid) was used instead of Tris–HCl and for pH 9, 9.5 and 10 10 mM CHES (*N*-cyclohexyl-2-aminoethanesulfonic acid) was used. The samples were incubated for 20 min at 1000 rpm at 25°C in an Eppendorf Thermomixer with an Eppendorf Smartblock™ 1.5 ml. After the incubation the beads were washed with the same experimental buffers once and then resuspended in counting buffer (10 mM Tris–HCl, pH 8.0, 1 mM EDTA, 200 mM NaCl, 0.2% SDS). The radioactive signal of DNA was quantified by liquid scintillation and was used for the calculation of protein DNA recovery (%) based on a reference sample containing the same amount of labeled ^32^P 685 bp DNA used in each sample. All DNA bridging experiments were performed at least in triplicate.

### Atomic force microscopy

Complexes of DNA and Rok protein were formed by incubating Rok with 50 ng of nicked pUC19 in AFM Buffer A (40 mM HEPES pH 7.9, 10 mM MgCl_2_, 60 mM KCl) at 37°C for 30 min. This mixture was then diluted 20-fold to a final buffer of 5 mM HEPES pH 7.9, 5.5 mM MgCl_2_, 3 mM KCl. 10 ul of the mixture was deposited onto a freshly cleaved mica disk. After 30 s, the mica disk was gently rinsed with 10 ml HPLC water, excess water on the surface was absorbed with lint-free tissue paper, and dried with filtered N_2_ gas. Images were acquired on a JPK Instruments NanoWizard 3 system in AC mode using a TESPA probe (Nanoworld) at a resonance frequency of 320 kHz. All images were captured at 512 × 512 square pixel resolution at a line rate of 1.5 Hz. The images were then processed using JPK Data Processing software. Images were flattened, and contrast was adjusted for clarity.

### Microscale thermophoresis

A serial dilution of the Rok or sRok protein was made from 32 to 0.250 μM using dilution buffer (20 mM Tris–HCl pH 8, 300 mM KCl, 10% glycerol, 0.1% Tween20 and 0.16 mg/ml acetylated BSA). Then the samples were diluted 1:1 with 80 nM DNA substrate in MilliQ water. This resulted in samples with a constant DNA substrate concentration of 40 nM with (s)Rok concentrations between 16 and 0.125 μM in 10 mM Tris–HCl pH 8, 150 mM KCl, 5% glycerol, 0.05% Tween20 and 0.08 mg/ml acetylated BSA. MgCl_2_ was added in dilution buffer when required. The samples were incubated for 5 min at room temperature and transferred to MST capillaries (Monolith NT.115 Premium Capillaries, NanoTemper, Germany). The measurement was done at 40% LED power and medium MST power using the NanoTemper Monolith NT.115. Total measurement time was 40 s, with 5 s laser off, 30 s laser on and 5 s laser off. Fnorm values were evaluated after 1.5 s of laser on. ΔFnorm values were calculated by subtracting Fnorm of DNA only. Occupancy values were calculated and fitted with a McGhee–von Hippel fitting algorithm assuming a binding site size (*n*) of 30 bp (27,34).

### Alphafold2 protein structure prediction

For the AlphaFold predictions MMseqs2 and LocalColabFold were run on the high performance computing facility ALICE at Leiden University (35–37). Multiple sequence alignments (MSAs) for Rok, sRok and Rok:sRok heterodimer were generated with MMseqs2 (38). Target databases used for these MSAs were constructed by the ColabFold team (https://colabfold.mmseqs.com/) and include UniRef30, BFD, Mgnify, MetaEuk, SMAG, TOPAZ, MGV, GPD and MetaClust2. The search sensitive parameter -s was set to 8. The constructed MSA was used as an input for LocalColabFold to predict dimer structures for Rok, sRok and Rok:sRok. No templates, 12 recycles, and AlphaFold-Multimer-v2 were used for these predictions. The structures were relaxed by AlphaFold's AMBER forcefield.

### His-tag pull down assay

40 μl of HisPur^TM^ Ni-NTA Magnetic beads (Thermo Scientific) per sample were pipetted into an Eppendorf tube. The buffer was exchanged for binding buffer (20 mM Tris–HCl pH 8.0, 500 mM KCl, 50 mM imidazole, 3.5% glycerol, 0.05% Tween, 0.8 mg/ml acetylated BSA) using a magnetic stand. 400 μl of the desired combination of Rok-6xHis and sRok was added to the bead suspension (final concentration in assay 8.75 μM) and incubated 30 minutes while mixing on an end-over-end rotator. The beads were washed once with binding buffer using a magnetic stand. The proteins were eluted with 25 μl of elution buffer (20 mM Tris–HCl pH 8.0, 500 mM imidazole, 3.5% glycerol) and incubated for 10 min with end-over-end rotation. The eluate was collected with a magnetic stand and 10 μl was mixed with 5 μl cracking buffer (50 mM Tris–HCl pH 6.8, 1% SDS, 25% glycerol, 1% β-mercaptoethanol, 0.05% bromophenol blue). 10 μl was loaded on a 4–15% mini-PROTEAN^®^ TGX™ Precast protein gel (Bio-Rad) and run at constant voltage (200 V) for 35 min. After the run, the gel was stained with 0.1% Coomassie Brilliant Blue in destain solution containing 40% ethanol and 10% acetic acid.

### *In vivo* complementation

*E. coli hns::kan* (strain NT135) cells were created by λ Red recombination (39). The cells were made chemically competent and transformed via heat shock with plasmid pRD408, pRD410, pRD412, pRD424 or an empty pUC19 vector. The transformed cells were plated on lysogeny broth (LB) agar (40) for growth curves, or on MacConkey agar (Sigma-Aldrich) with 0.4% salicin (Sigma-Aldrich) or bromothymol blue (Sigma-Aldrich) indicator plates with 0.5% salicin, all with 50 μg/ml kanamycin and 75 μg/ml ampicillin and incubated overnight at 37°C.

For growth curve measurements, liquid starter cultures were prepared by inoculating LB medium with the appropriate antibiotics with single colonies from LB agar plates. Cultures were grown until an OD_600_ of 0.8–1.0. Next day, all cultures were diluted to an OD_600_ of 0.01. Next, the OD_600_ value of each culture was measured at regular time intervals during growth at 37°C, 200 rpm. After finishing the growth curve, the plasmids were isolated using the Thermo Scientific GeneJET Plasmid Miniprep Kit and next checked with DNA Sanger sequencing (BaseClear) to rule out the occurrence of mutations.

### Construction of *B. subtilis* strains

All constructed *B. subtilis* strains are derivatives of strain 168. Cassettes allowing the controlled expression of *rok*, *srok* or *srok + rok* were placed at the *amyE* locus of the *rok* deletion strain PKS21 (Supplementary Table S1) as follows. Genes *srok* and *rok* were amplified by PCR using as template total DNA isolated from strain PKS11 harboring pLS20 in combination with primer sets [oAND314/oAND315] and [oAND316/oAND317], respectively. The PCR products were digested with HindIII and SalI, or SalI and NheI (New Engeland Biolabs, USA) and then cloned in vector pDR110 (a gift from D. Rudner) digested with the same enzymes to produce pAND520 and pAND521. Plasmid pAND522 was generated cloning the fragment of *rok* behind the srok gene on plasmid pAND520. Plasmid pDR110 is a *B. subtilis amy*E integration vector that contains a multiple cloning site located behind the IPTG-inducible P*_spank_* promoter. Next, plasmids pAND520, pAND521 and pAND522 were used to transfer competent PKS21 cells and selecting for spectinomycin resistant transformants. *B. subtilis* competent cells were prepared as described (41). Double cross over events of the resulting strains AND520, AND521 and AND522 were confirmed by the loss of a functional amylase gene.

### RNA-seq

*B. subtilis* strains were grown in Lysogeny broth (LB) medium (40) or on 1.5% LB agar plates supplemented with spectinomycin (100 μg/ml). For total RNA extraction, the bacteria were grown in liquid media with shaking overnight (ON) at 37ºC and diluted 1/100 times in fresh LB media with 1 mM of IPTG. At OD_600_ = 0.8–1, 1.5 ml of the culture was harvested by centrifugation and stored at −80ºC. Pelleted cells were thawed on ice and then lysed using a bead mill. Total RNA was isolated using Monarch^®^ Total RNA Miniprep Kit (New England Biolabs, USA) and stored at −80ºC.

Concentrations of RNA samples were measured using the Nanodrop, and 500 ng was loaded on a 1% agarose bleach gel to verify the quantity and quality. The RiboCop rRNA depletion kit (Lexogen Vienna, Austria) was used to remove ribosomal RNA from 500 ng total RNA. Subsequently, the CORALL Total RNA-SeqTotal RNA Library Kit (Lexogen Vienna, Austria) was used to prepare the library preps for Illumina sequencing. Samples were sequenced on the Illumina NextSeq 1000 to generate 100 bases single end reads (100SE) with an average read-depth of 8–12M reads per sample. The quality of the resulting fastq reads we checked using FastQC v0.11.9 (Babraham Bioinformatics, Cambridge) and mapped on the reference genome (*Bacillus subtilis subsp. subtilis str. 168* (GenBank identifier AL009126.3)) using Bowtie2 v2.4.2 using default settings (42). Resulting SAM files were converted to BAM using SAMtools 1.11 and featuresCounts 2.0.1 was used to obtain the gene counts for each gene annotated in the reference genome taking into account the orientation of genes and reads (43,44). Duplicate samples were summarized by calculating the mean counts for each gene.

### RNA-seq data processing and analysis

To calculate the effect of ectopic induction of *rok*, *srok* or *rok* + *srok* on expression on the *B. subtilis* genes, the average of normalized counts obtained for the Δ*rok* strain PKS21 were subtracted from the normalized counts derived from wild type, AND520, AND521 and AND522 strains. Note that all strain were grown in the presence of IPTG, which avoids differential gene profile effects due to IPTG. These differences were transformed by taking their square root. Plots showing differential gene expression profiles as a function of genome position were generated using the gnuplot tool (www.gnuplot.info). For comparison, such plots were also generated using previously published data of DNA binding of Rok determined by ChIP and DNA coverage techniques (16,23). We next fitted several distributions to the aggregated values from differences, which revealed that our data were best modelled by the logistic distribution, and therefore we used this distribution to identify differentially expressed genes (DEGs). These exercises were done using R (Foundation for statistical computing (https://www.R-project.org/)) in combination with the package ‘fitdistrplus’ (45) obtaining the following parameters: location = 0.0715, scale = 0.66927.

From this distribution, the associated *P*-value for every gene in each condition was transformed into a *q*-value that incorporates a false discovery rate-based multiple testing correction, using the ‘qvalue’ package for R (https://bioconductor.org/packages/release/bioc/html/qvalue.html). Those genes that exhibit an associated *q*-value < 1E−3 were selected as DEGs.

The list of DEGs from each strain was uploaded to the FUNAGE-Pro (FUNctional Analyis and Gene set Enrichment for Prokaryotes) server (46), to detect enrichment in operons and KEGG pathways (47).

### Analysis of Rok and sRok sequences

The NCBI protein database was used to search for all sequences annotated as Rok. The resulting sequences were divided based on sequence length: ≥190 aa for Rok and ≤189 aa for sRok as previously suggested (24). Also, sequences NP_389307.1 (Rok) and BAJ76946.1 (sRok) were used for BLAST searches, but no additional candidate Rok proteins were identified. Rok sequences from species with or without sRok present on the chromosome were gathered with Batch Entrez and aligned using EMBOSS Clustal Omega using default parameters (48). Sequence logos were generated using Skylign (https://skylign.org) using default parameters (49).

## RESULTS

### Rok compacts DNA

**Figure 1.**
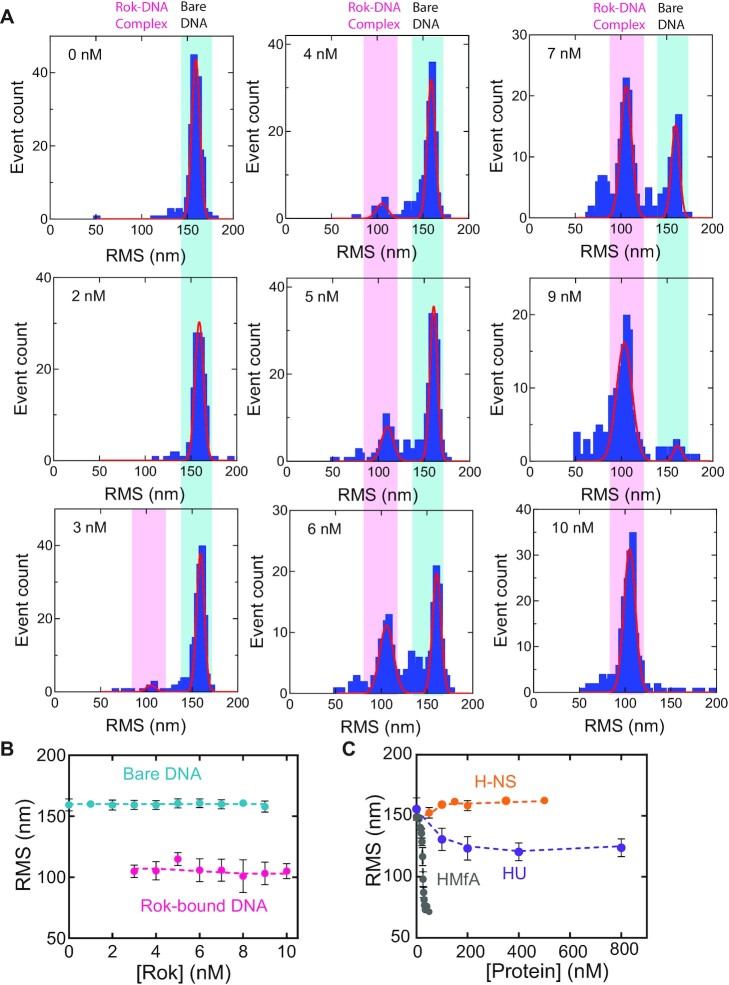
*B. subtilis* Rok compacts DNA. (**A**) Histograms of root mean square displacement (RMS) obtained for 32%GC DNA as a function of Rok at concentrations of 0, 2, 3, 4, 5, 6, 7, 9 and 10 nM as measured by TPM in the presence of 50 mM KCl. The histograms were fitted to Gaussian distributions, in which the RMS value at ∼150 nm represents bare DNA and the population with an RMS at ∼100 nm represents DNA bound by Rok. The bare DNA and Rok–DNA complex populations are highlighted with a light blue and magenta box, respectively. The data for each concentration originates from at least two independent measurements. (**B**) RMS values obtained for 32% GC DNA as a function of Rok at concentrations from 0 nM to 10 nM. Blue and magenta dots represent the average RMS resulting from fitting with a Gaussian distribution, where blue represents bare DNA and magenta Rok–DNA complexes, respectively. Error bars represent the propagated standard deviation from at least two independent measurements. Due to their small size, some error bars are hidden behind the data points. How the DNA tethers are distributed between the two populations is not taken into account in this representation. Dashed lines are lines to guide the eye. (**C**) RMS as a function of protein concentration of *E. coli* H-NS and HU, and *M. fervidus* HMfA. Data were taken from van der Valk *et al.* (H-NS) (27), Driessen *et al.* (HU) (97) and Henneman *et al.* (HMfA) (54). Error bars represent the standard deviation; due to their small size they are hidden behind the data points. Dashed lines serve as lines to guide the eye.

To determine the architectural properties of Rok, we investigated the effect of Rok binding on the conformation of DNA using Tethered Particle Motion (TPM) (30,32). In TPM experiments the Root Mean Square displacement (RMS) of a bead (exhibiting thermal motion) at the extremity of a DNA substrate attached to a glass surface, provides a readout of DNA conformation. Whereas an increase in RMS following the binding of proteins is indicative of DNA stiffening, an RMS reduction reflects DNA softening, binding or bridging upon protein binding. We investigated the interaction between Rok and an AT-rich (32% GC) DNA substrate, which we used earlier to study the DNA-binding properties of H-NS (27) and MvaT (28,50). We determined the effect of Rok on DNA conformation by titration from 0–10 nM (Figure 1A). Bare DNA had an RMS of 159 ± 2 nm. Upon addition of 3 nM Rok, a second population at an RMS of ∼105 nm appeared. Saturation of Rok binding was achieved at 10 nM; at this concentration only the population with reduced RMS was observed (Figure 1A and B). At several concentrations two extra minor populations can be observed at an RMS of ∼125 and ∼80 nm. As these populations were not consistently present (and therefore do not represent a main Rok–DNA complex) and could not be fitted due to low occupancy, they were not taken into account for occupancy calculations and further analysis (see below). The observed reduction of RMS implies that Rok does not form DNA stiffening filaments along DNA as observed for other H-NS-family proteins under similar conditions (51–53), but instead indicates that binding of Rok compacts the DNA (Figure 1B). However, it cannot be ruled out that a Rok filament is formed that effectively shortens the DNA, as has been found for HMf proteins (Figure 1C) (54). The compaction of the DNA would then be due to DNA bending induced by binding of individual Rok proteins in a filament. The reduction in RMS might also be attributed to DNA bending without filament formation as observed for HU (Figure 1C) (55) or to DNA bridging. The fact that compaction occurred at low protein concentration and that the structural transition was abrupt suggests cooperative behavior.

### Rok is able to bridge DNA

**Figure 2.**
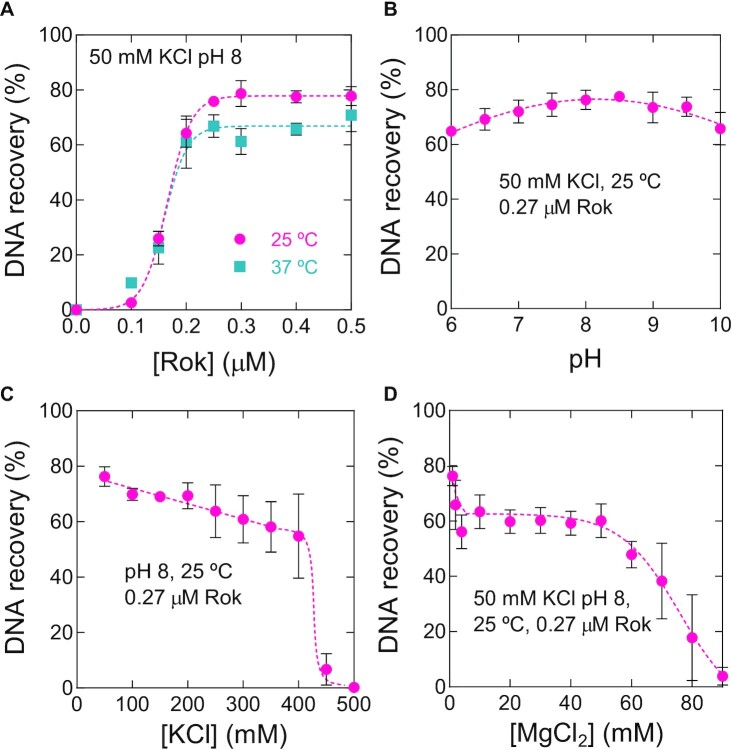
Rok exhibits DNA bridging activity, which is only mildly affected by temperature, pH and salt concentration. (**A**) DNA recovery (as a percentage of the input DNA) as a function of Rok concentration from 0 to 0.5 μM as measured using the DNA bridging assay in the presence of 50 mM KCl at 25ºC (red) and 37ºC (blue), respectively. (**B**) DNA recovery as a function of pH from 6 to 10 in the presence of 0.27 μM Rok at 25ºC. (**C**) DNA recovery as a function of KCl concentration from 35 to 500 mM in the presence of 0.27 μM Rok at 25ºC. (**D**) DNA recovery as a function of MgCl_2_ concentration from 1 to 90 mM in the presence of 0.27 μM Rok at 25ºC. Data are plotted as mean values of three independent measurements and the error bars represent the standard deviation. Dashed lines serve as lines to guide the eye.

The DNA compaction observed in the TPM experiments described above could have its structural basis in DNA bridging, which is supported by the fact that Rok can induce chromosomal loops (23). This led us to hypothesize that Rok, like H-NS, is able to bridge DNA. We therefore investigated the ability of Rok to bridge DNA in a quantitative biochemical DNA bridging assay, which we used earlier to evaluate the effect of altering physico-chemical conditions on the DNA bridging efficiency of H-NS and MvaT (27,28). In this assay, biotinylated DNA bound to streptavidin-coated magnetic beads is used as bait in combination with ^32^P-labeled prey DNA offered *in trans* that can be recovered by magnetic pull-down of beads when bridged by protein. The radioactive signal of the DNA pulled down is a proxy of DNA bridging efficiency. The DNA used in the bridging assay was the same as that used in TPM experiments. The TPM and bridging experiments are fundamentally different in the sense that they interrogate DNA at single molecule and bulk DNA level respectively, which is why higher protein concentrations are needed in the bridging assay (56). To determine whether Rok bridges DNA we carried out a titration with Rok from 0–0.5 μM. In the absence of Rok, no radioactive DNA was recovered. DNA recovery increased with increasing Rok concentrations. Saturation of DNA recovery occurred at a Rok concentration of 0.3 μM (Figure 2A). These data unambiguously show that Rok is a DNA bridging protein, which we confirmed by AFM imaging (Supplementary Figure S1). Rok has a similar DNA bridging efficiency as H-NS at 25ºC, yet reaches this efficiency at 10 times lower concentration (0.3 μM versus 3 μM (27)), which we attribute to the high DNA binding cooperativity of Rok. The minimal length of DNA that Rok could bridge was 100 bp (Supplementary Figure S2), which suggests that multiple Rok dimers are needed to form a stable bridge. During natural transformation, a process in which Rok has been implicated (18), dsDNA is processed into ssDNA and only a single DNA strand (ssDNA) is absorbed. Therefore, we tested if Rok might be able to bridge incoming ssDNA with genomic dsDNA and therewith play a possible role in recombination. However, Rok was unable to bridge prey ssDNA with dsDNA bait (Supplementary Figure S2). Some bridging was observed with a 685 bp ssDNA substrate, but this was most likely due to binding of Rok to dsDNA structures generated by internal hybridization as we were unable to reproduce these observations using poly(A) ssDNA. As internal hybridization of ssDNA can also occur inside the cell, Rok might still play a role in recombination by bridging small DNA structures to genomic DNA.

### DNA bridging activity of Rok is only mildly sensitive to environmental conditions

Bacteria adapt to environmental changes and environmental cues are known to have a direct effect on the function of H-NS-like proteins (51,57–60). *B. subtilis*, found in soil and the gastrointestinal tract of livestock and humans, is exposed to rapid changing conditions, which requires an ability to adapt to different environmental conditions via changes in transcription of specific genes. To study if Rok plays a direct role in this response by responding to environmental changes through altering its binding (mode) at Rok-regulated genes as seen for H-NS (57–59,61,62), we tested the effect of various physico-chemical conditions on Rok's DNA bridging efficiency.

First, we investigated the effect of temperature and pH. An increase in temperature from 25ºC to 37ºC caused a slight drop in DNA recovery from 80 to 60% in DNA bridging assays (Figure 2A). DNA was also rather efficiently recovered over a pH range from 6 to 10 (Figure 2B). Strikingly, even crossing the pI of Rok (9.31) did not interfere with its capacity to bridge DNA.

Besides temperature and pH, *B. subtilis* is frequently challenged to adapt to osmotic up- and downshifts in its natural habitat (63,64). To determine whether Rok's DNA bridging activity is osmo-sensitive, we investigated the effect of changing concentrations of monovalent and divalent cations. An increase in concentration of KCl from 50 to 300 mM had no significant effect on the DNA bridging activity of Rok (Figure 2C). A mild decrease in DNA recovery from 80 to 60% was observed when increasing the MgCl_2_ concentration from 0 to 20 mM and this remained constant until 60 mM (Figure 2D). This result might indicate that the Mg^2+^ concentration modulates the affinity of Rok for DNA as has been previously suggested for H-NS (65). To check if this is the case, we performed microscale thermophoresis (MST) experiments with Rok and a 78 bp DNA substrate. Without MgCl_2_ an apparent binding affinity (*K*_D_) of 79.4 ± 20 μM was obtained (Supplementary Figure S3). The affinity improved slightly in the presence of 10 mM MgCl_2_ (*K*_D_ of 34.6 ± 8.0 μM), but it decreased substantially with MgCl_2_ concentrations above 25 mM (*K*_D_ of around 800 μM). We concluded that Rok exhibits the highest DNA binding affinity at 10 mM MgCl_2_ in contrast to what has been previously observed for H-NS. Thus, instead of decreased DNA binding observed for H-NS, Rok displayed increasing DNA binding activities at 5–20 mM MgCl_2_. We also tested a specific DNA substrate, which differs from the aspecific substrate by having a specific Rok binding site (TACTA) present in the middle, which was found previously to be one of the most favorable for Rok binding (66). We observed similar behavior as for the aspecific DNA substrate, but Rok-binding remained in the high affinity regime of *K*_D_ ∼ 100 μM over a wider range of MgCl_2_ concentration (up to 35 mM MgCl_2_ instead of 25 mM) before transitioning to the low-affinity regime (*K*_D_ ∼ 800 μM) (Supplementary Figure S3).

The intracellular concentrations of K^+^ and Mg^2+^ in *B. subtilis* have been determined to be 27 ± 10 mM and 1–2 mM, respectively (67). Figure 2C and D shows that DNA recoveries are at maximum levels at these cation concentrations; the decrease in DNA recoveries observed above 400 and 50 mM concentrations of KCl and MgCl_2_ respectively are well above the physiological concentrations and hence are unlikely to be relevant under natural conditions. We attribute the reduction in DNA recovery at high cation concentration to complete disintegration of DNA–Rok–DNA bridged complexes, associated with the reduction in DNA binding affinity, rather than a switch from a Rok–DNA bridge to a Rok–DNA nucleofilament. Unlike H-NS and MvaT (27,28), the formation of bridged Rok–DNA complexes also does not require a particular concentration of monovalent (K^+^) or divalent (Mg^2+^) cations. These observations highlight that DNA bridging activity of Rok neither requires KCl or MgCl_2_ for bridging, nor is strongly inhibited by these ions at biologically relevant concentrations. To examine if Rok might be able to induce liquid-liquid phase separation (LLPS), 1,6-hexanediol, a commonly used alcohol to dissolve LLPS assemblies (68–70 and references therein), was added to the bridging assay before Rok–DNA bridge formation. DNA was efficiently recovered up to 5% 1,6-hexanediol (Supplementary Figure S4), suggesting that Rok does not form LLPS assemblies.

Taken together, these observations indicate that the DNA bridging activity of Rok is only mildly affected by changes in physico-chemical conditions. This is unexpectedly different from H-NS and MvaT where much larger effects were observed (27,28,53,57).

### The neutral linker of Rok has a role in DNA binding cooperativity

**Figure 3.**
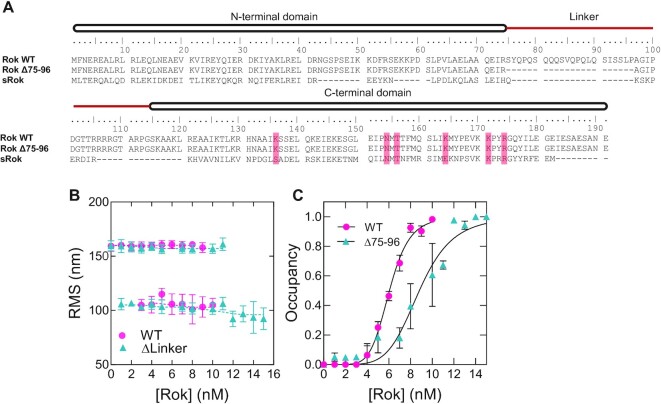
The linker domain of Rok is important for its cooperative DNA binding. (**A**) Sequence alignment of Rok wildtype (NCBI accession number: NP_389307.1), Rok Δ75–96 and sRok (NCBI accession number: YP_004243533.1). The N- and C-terminal domain are indicated with a black box and the linker domain with a red line. The residues important for DNA binding are highlighted in red. (**B**) RMS values obtained for 32% GC DNA as a function of Rok Δ75–96 concentration as measured by TPM in the presence of 50 mM KCl. The RMS values were determined from fitting with a Gaussian distribution. For reference, Rok WT data is shown (reproduced from Figure 2B). Error bars represent the propagated standard deviation from at least two independent measurements. (**C**) DNA occupancy (fraction of Rok-bound tethers of the total amount of DNA tethers) as function of protein concentration in nM. The data points were fitted using the Hill binding model.

Previously, we found that H-NS-like proteins have a conserved asymmetrical charge distribution, with the N-terminal domain mainly negatively charged and the linker and C-terminal domain positively charged (8). This asymmetrical charge distribution is needed for interdomain interactions which are characteristic for nucleoprotein filament formation (28,71). Rok is a notable exception with a less pronounced charge distribution and a neutral linker (8). Therefore, we proposed that Rok cannot form a nucleoprotein filament because of weaker interdomain interactions. We attempted to test if the introduction of charges could result in DNA stiffening behavior as observed for H-NS (Figure 1C). Unfortunately, a recombinant Rok variant in which the neutral part of the linker was replaced with the (charged) H-NS linker was present in the insoluble fraction after cell lysis (data not shown). Next, we investigated whether removal of Rok's neutral amino acids (residues 75–96) would allow the recombinant protein Rok Δ75–96 to form nucleofilaments (Figure 3A). Although, this Rok variant could successfully be expressed and purified, TPM experiments with the Rok Δ75–96 variant revealed the same level of DNA compaction as observed for wild type Rok (Rok WT Figure 3B and Supplementary Figure S5A). This strongly indicates that removal of the neutral linker in Rok does not promote DNA stiffening and that hence this Rok variant does not form filaments upon DNA binding.

Removal of the linker however affected DNA binding cooperativity, as evident from the less steep increase of DNA occupancy for the Rok Δ75–96 variant compared to the wild type protein (Figure 3C). When fitted to the Hill equation, affinities for Rok WT and the Rok Δ75–96 variant were 6.1 ± 0.07 and 8.8 ± 0.3 nM respectively and the Hill coefficients (*n*) were 6.5 ± 0.4 for Rok wildtype and 5.7 ± 0.9 for the Rok Δ75–96 variant. Similar behavior was observed for the Rok Δ75–96 variant in the DNA bridging assay: it can bridge DNA with similar efficiency as Rok wildtype, but with the transition from low to high DNA recovery occurring over a wider concentration range: between 0.1–0.3 μM for Rok wildtype and between 0.1–0.5 for Rok Δ75–96. (Supplementary Figure S5B). Together, these results show that the deletion of the neutral linker in Rok does not affect DNA filament formation and bridging, but that it affects the cooperativity of DNA binding.

### sRok exhibits nucleoprotein filament formation and can modulate the DNA bridging activity of Rok

Besides the artificial Rok Δ75–96 variant, a number of *Bacillus* species and strains encode a variant of Rok that lacks the linker region. We refer to this variant as small Rok (sRok) (8) (Figure 3A). sRok was first identified on the large conjugative *B. subtilis* plasmid pLS20 (24). It was shown to associate with the host chromosome and to be able to replace Rok as regulator in the competence pathway (24). Therefore, we expected that Rok and sRok would have similar DNA binding properties, while the sensitivity to the environment may differ due to the lack of a linker in sRok.

**Figure 4.**
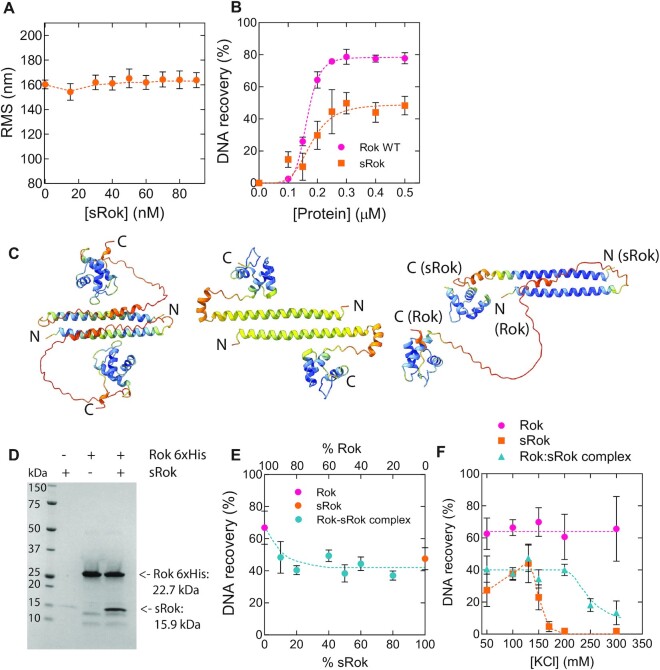
The bridging activity of sRok can be modulated by salt concentration. (**A**) RMS values obtained for 32% GC DNA as a function of sRok concentration as measured by TPM in the presence of 50 mM KCl. The RMS values were determined from fitting with a Gaussian distribution. Error bars represent the propagated standard deviation from at least two independent measurements. Some error bars are hidden behind the data points. (**B**) DNA recovery (%) as function of (s)Rok concentration in the presence of 50 mM KCl at 25°C. For reference, Rok WT is shown (reproduced from Figure 2A). Data are plotted as mean values from three independent measurements and the error bars represent the standard deviation. Dashed lines serve as lines to guide the eye. (**C**) Structural predictions using Alphafold of Rok homodimer, sRok homodimer and Rok:sRok heterodimer (left to right). The protein structures are colored by the predicted DT-Cα (PLDDT) values with the following color scheme: blue (>90), light blue (90–70), yellow (70–50), orange (<50). The PLDDT indicates the local confidence in the predicted structures from 0–100, with 100 corresponding to highest confidence. (**D**) SDS-PAGE analysis of His-tag pull down assay. Rok 6xhis was captured on HisPur™ Ni-NTA Magnetic beads and, when applicable, sRok was added in 1:1 molar ratio. (**E**) DNA recovery (%) measured in the presence of 50 mM KCl + 1 mM MgCl_2_ at 25°C with different ratios Rok:sRok. The total amount of protein used was constant at 0.5 μM. (**F**) DNA recovery (%) measured in the presence of 1 mM MgCl_2_ at 25°C and 0.5 μM protein with different KCl concentrations. Dashed lines serve as lines to guide the eye.

To test this, we investigated the DNA binding properties of purified sRok in TPM assays using the same DNA fragment as for Rok in Figure 1. The experiments revealed that the RMS of the DNA tether was only mildly—if at all—affected by protein concentration (Figure 4A). While this could point at low DNA binding affinity or non-functional protein, DNA bridging experiments (see below) demonstrate that the protein binds DNA. These observations suggest that sRok binds DNA without affecting DNA conformation, which is in sharp contrast to the DNA compaction observed upon Rok binding (Figure 1A). In the bridging assay, sRok reached its maximal DNA recovery in the same concentration range as Rok (Figure 4B).

We considered that Rok might be functionally modulated by sRok, analogous to the modulation of DNA bridging efficiency of H-NS and other H-NS-like proteins by their protein-partners (8). Rok has been shown to dimerize via its N-terminal domain (66). The possibility that Rok and sRok hetero(di)merize is realistic considering the high level of conservation between the N-terminal multimerization domain of Rok and sRok (8). Using Alphafold2 we predicted the structures of Rok and sRok homodimers and of Rok-sRok heterodimers (Figure 4C) with high confidence levels (Supplementary Figure S6A–C). Both proteins exhibit a similar tertiary structure and dimerize using their N-terminal helix (residues 1–47). At the monomer-monomer interface, the two α-helices interact in a coiled-coil-like manner and form similar salt bridges and hydrophobic interactions in all three predicted structures (Supplementary Figure S6D). We also attempted to predict higher order structures to gain insight in the oligomerization interface, but Alphafold2 was unable to generate a confident model. To test whether the predicted heteromerization indeed can occur we generated a Rok variant with a 6xHis-tag at the C-terminus that we exploited for pulldown using HisPur™ Ni-NTA Magnetic beads (see Materials and Methods). Figure 4D shows that sRok lacking a His-tag was pulled down together with Rok-6xHis confirming heteromerization of the two proteins. Next, we performed bridging assays using different ratios of Rok and sRok (Figure 4E). Interestingly, the presence of only 20% of sRok in a Rok:sRok mixture was sufficient to lower the DNA recovery to about 40%, similar to the DNA recovery of sRok alone. This latter result not only supports that Rok and sRok can form heterodimers, but also shows that sRok can modulate the DNA bridging efficiency of Rok. To investigate further the interplay between Rok and sRok we studied the effects of salt concentration on the DNA bridging efficiency of sRok and the Rok:sRok complex. For this, bridging experiments were performed using different KCl concentrations for both sRok and a 1:1 Rok:sRok complex. While Rok was only mildly sensitive to these changes (Figures 2 and 4F), DNA bridging by sRok peaked around 130 mM KCl, but became strongly inhibited at higher KCl concentrations (Figure 4F). MST also showed that sRok is less tolerant than Rok to increasing MgCl_2_ concentrations (Supplementary Figure S7). At 20 mM MgCl_2_ or higher the binding was too weak to fit a *K*_D_ value. Also, the presence of a specific high-affinity DNA sequence for Rok yielded no improvement in sRok binding. The Rok:sRok complex showed intermediate behavior with a constant DNA bridging efficiency up to 200 mM KCl (Figure 4F). These results show that - in contrast to Rok - sRok-mediated DNA bridging is osmo-sensitive and that to a lesser extent this also translates to the Rok:sRok complex. These results open up the possibility of controlling Rok-mediated gene repression via protein partners such as sRok.

### Rok and sRok cannot complement the absence of *hns* in *E. coli*

The results in Figure 4 might suggest that sRok is functionally more similar to H-NS than Rok due to its responsiveness to changes in osmolarity. To test this hypothesis, we performed *in vivo* complementation experiments in *E. coli*. Previously, this approach showed that MvaT and Lsr2 can complement the absence of H-NS *in vivo* (72,73). We used *E. coli hns::kanR* (NT135) and transformed it either with an empty pUC19 vector or with a pUC19-derived vector containing the *hns* promoter followed by the respective protein coding sequence. One of the best-characterized operons that is repressed by H-NS is the *bgl* operon (74). Expression of this operon, which results in the uptake and fermentation of aryl-β-D-glucosides (75), has been used to test whether H-NS variants or potential H-NS like proteins can complement the *hns* knockout phenotype. The pH difference caused by either the ability (acidic pH) or the inability (basic pH) to ferment an aryl-β-D-glucoside can be visualized on MacConkey agar or bromothymol blue indicator (BTB) plates supplemented with salicin (73,76,77). As expected, *E. coli* NT135 harboring the empty plasmid was able to use salicin as carbon source resulting in red and yellow colonies on MacConkey (Supplementary Figure S8A) and BTB agar plates (Supplementary Figure S8B) respectively. *E. coli* NT135 ectopically expressing *hns* from the plasmid (containing the *hns* gene) cannot use salicin and grow on peptone. They formed yellow and blue colonies on MacConkey (Supplementary Figure S8A) and BTB plates (Supplementary Figure S8B), respectively. When transformed with pRD424 (Rok), the colonies were red on MacConkey agar, sometimes with a yellow halo around them and yellow on BTB plates, indicating that Rok represses the *bgl* operon at most only partly. Different results were obtained for sRok; the ectopic expression of *srok* resulted in formation of yellow colonies on MacConkey plates, indicating that the *bgl* operon is repressed in these cells. However, on BTB plates also yellow colonies were obtained indicating that the *bgl* operon is expressed. This discrepancy might be explained by partial repression of the *bgl* operon and different sensitivities of the pH indicator used in the respective plates. Finally, cells expressing *rok Δ75–96* formed yellow and brown/green colonies on MacConkey and BTB plates, respectively. This indicates that Rok Δ75–96 can repress the *bgl* operon to a better extent than Rok and sRok, but cannot achieve the complete repression caused by H-NS.

The (dis)ability of Rok, sRok and Rok Δ75–96 to complement H-NS *in vivo* was further analyzed by studying possible reversion of growth defects observed for the *Δhns* strain NT135. Growth curves of *E. coli* NT135 expressing *hns, rok, srok* or *rok Δ75–96* are shown in Supplementary Figure S8C. Expression of either Rok or sRok did not restore the growth defect caused by the lack of H-NS; in fact, their expression aggravated the growth defect caused by the lack of H-NS. This indicates that both Rok and sRok cannot bind to and repress most of H-NS-regulated genes but instead may bind to non-H-NS-regulated genes, which could explain the detrimental effect of (s)Rok on cell growth. Another, not mutually exclusive, possibility is that (s)Rok binds (most) H-NS-regulated genes, but exhibits a different mode of DNA binding, causing aberrant gene expression. Interestingly, cells expressing *rok Δ75–96* grew at the same rate as the positive H-NS control, indicating complementation of the *Δhns* growth defect by Rok Δ75–96. This suggests substantial occupation by the Rok Δ75–96 variant of genomic sites otherwise bound by H-NS with regulatory function or key function in transcriptional regulation or structural organization.

### Rok and sRok exert different effects on transcription in *B. subtilis*

The *in vitro* results presented above demonstrate that while Rok and sRok are both able to bridge DNA, they exhibit different physico-chemical behaviors. Due to these differences, the two proteins may exert distinct effects on transcription. Upon heterologous expression in *E. coli* we indeed observed different abilities of the proteins to complement H-NS in a *Δhns* strain. We next directly investigated the possibility that Rok and sRok have distinct effects on transcription in *B. subtilis* using an RNAseq approach. First, we generated a *rok* null mutant (Δ*rok*) and used this strain to construct derivatives containing a cassette at the *amyE* locus having a copy of s*rok* or *rok* under the control of the IPTG-inducible promoter P*_spank_* (strains AND520 and AND521, respectively). In addition, we constructed a strain (AND522) in which P*_spank_* drives the expression of both *srok* and *rok*. Total RNA was isolated from these and control strains grown under the same conditions in the presence of 1 mM IPTG. After processing, the RNA samples were used to generate cDNA libraries using a “directional RNAseq” procedure that preserved information about a transcript's direction. The generated libraries were subjected to Illumina sequencing to generate 100-nt fragments, and those that passed quality controls (see Material and Methods) were used to calculate the apparent expression level of individual genes.

**Figure 5.**
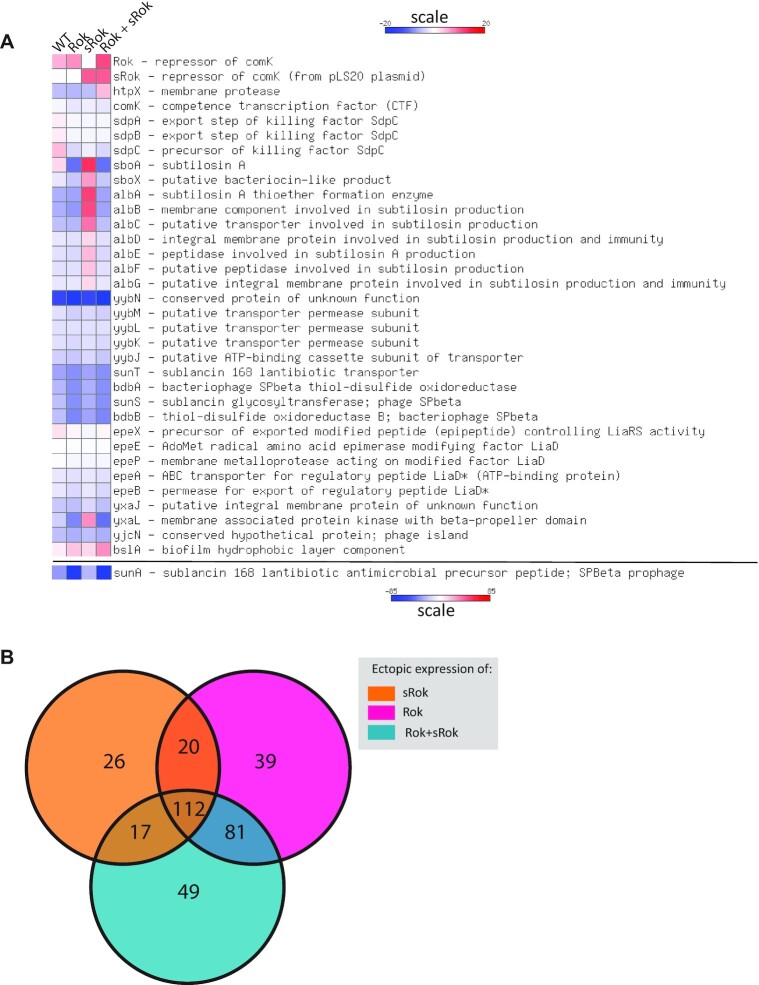
Effects of Rok, sRok and Rok + sRok on the expression of *B. subtilis* genes. (**A**) Heat map representations of a set of known Rok-regulated genes in response to *rok*, *srok* and *rok + srok* expressed from an ectopic promoter. The heat map as a response to *rok* expressed from its native promoter is included as a control. Changes in expression observed for *sunA* in response to the different *rok* variants exceeded the −20 to +20 range. Therefore, for clarity, this gene was plotted separately using a larger range. Blue and red color reflect lower and higher expression levels with respect to the *Δrok* strain. (**B**) Venn diagram of differentially expressed *B. subtilis* genes when *rok*, *srok* or *rok + srok* are ectopically expressed in an otherwise isogenic background. The number of DEG are indicated for each of the three different conditions: ectopic expression of *rok*, *srok* or *rok + srok*. Numbers in the intersections correspond to DEG shared by the corresponding different conditions.

Several genes known to be repressed by Rok are given in Figure 5A and Supplementary Table S5 that also lists the effect of ectopic expression of Rok in a Δ*rok* background on these genes observed in our studies. The table shows that under these conditions *rok* was expressed 2-fold higher from the P*_spank_* promoter as compared to its native promoter. Importantly, the observation that most of these reported Rok-regulated genes were also repressed in our experiments in which *rok* was expressed form its native or the P*_spank_* promoter validates our approach (Figure 5A, Supplementary Table S5). The exceptions for which the expression was not or only slightly affected in our experiments can be explained by the differences in growth conditions in the different studies. For instance, our samples were taken at late exponential growth phase from cultures growing in rich LB medium when *comK* and the *sdp* operon are expressed at only very low levels. Remarkably, ectopic expression of *srok* or *srok* + *rok* affected several of these genes differently compared to *rok*. For example, *sboA* was down- and upregulated by Rok and sRok, respectively, and *htpX* was upregulated in the presence of both Rok + sRok, but downregulated by each of the two Rok variants individually. Thus, the two highly related transcriptional regulators (48% sequence similarity for the total proteins, 55% similarity for the DNA binding domains) do not seem to target an identical set of genes. Moreover, the Rok variants seem to affect each other's role in transcription regulation. To study the possibility that besides the small set of selected genes mentioned above also other genes are differentially regulated by Rok, sRok and Rok + sRok, we plotted the expression levels along the entire genome for each of these three overexpressing strains with respect to the Δ*rok* strain (Supplementary Figure S9). These plots show that Rok, sRok and Rok + sRok affect the expression at multiple different loci along the entire *B. subtilis* chromosome and confirm therefore that their regulatory effect is not identical. In line with the results presented in Figure 5A, these plots also show that the expression profile observed for simultaneous expression of Rok and sRok is distinct to those observed for the individual Rok variants. The genome wide expression data were then used to select statistically differentially expressed genes (DEG) (qvalue < 1e-3). This resulted in 175, 252 and 259 DEG for conditions in which sRok, Rok and Rok + sRok were overexpressed, respectively (Supplementary Tables S6, S7 and S8). These numbers correspond to about 5% of all protein-encoding *B. subtilis* genes. To gain insight into (dis)similarities between the DEG of the three experimental conditions they were presented as Venn diagram (Figure 5B) that provided the following information. First, different subsets of DEG were regulated by both Rok and sRok (132 genes [38.4%]), or by Rok + sRok with either Rok (193 genes [56.1%]) or sRok (129 genes [37.5%]). Second, about one third of the DEG was regulated under each of the three different conditions (112 genes [32.6%]). Thus, as might have been expected for two highly related proteins, substantial overlap in DEG were observed for Rok, sRok and Rok + sRok. However, as already hinted at by the analysis of a small number of genes (Figure 5A), the sets of genes regulated by Rok, sRok and Rok + sRok are not identical. Besides considerable subsets of DEG being affected by only Rok (120 genes, [34.8%]) or sRok (43 genes, [12.5%]), a substantial subset of DEG was affected by only Rok + sRok (49 genes, [14.2%]). Particularly this latter observation strongly suggests that the regulatory capacity of Rok is influenced by sRok and *vice versa*. Importantly, these results are in line with the *in vitro* data presented above which show that the presence of sRok affects the DNA binding properties of Rok (Figure 4).

**Figure 6.**
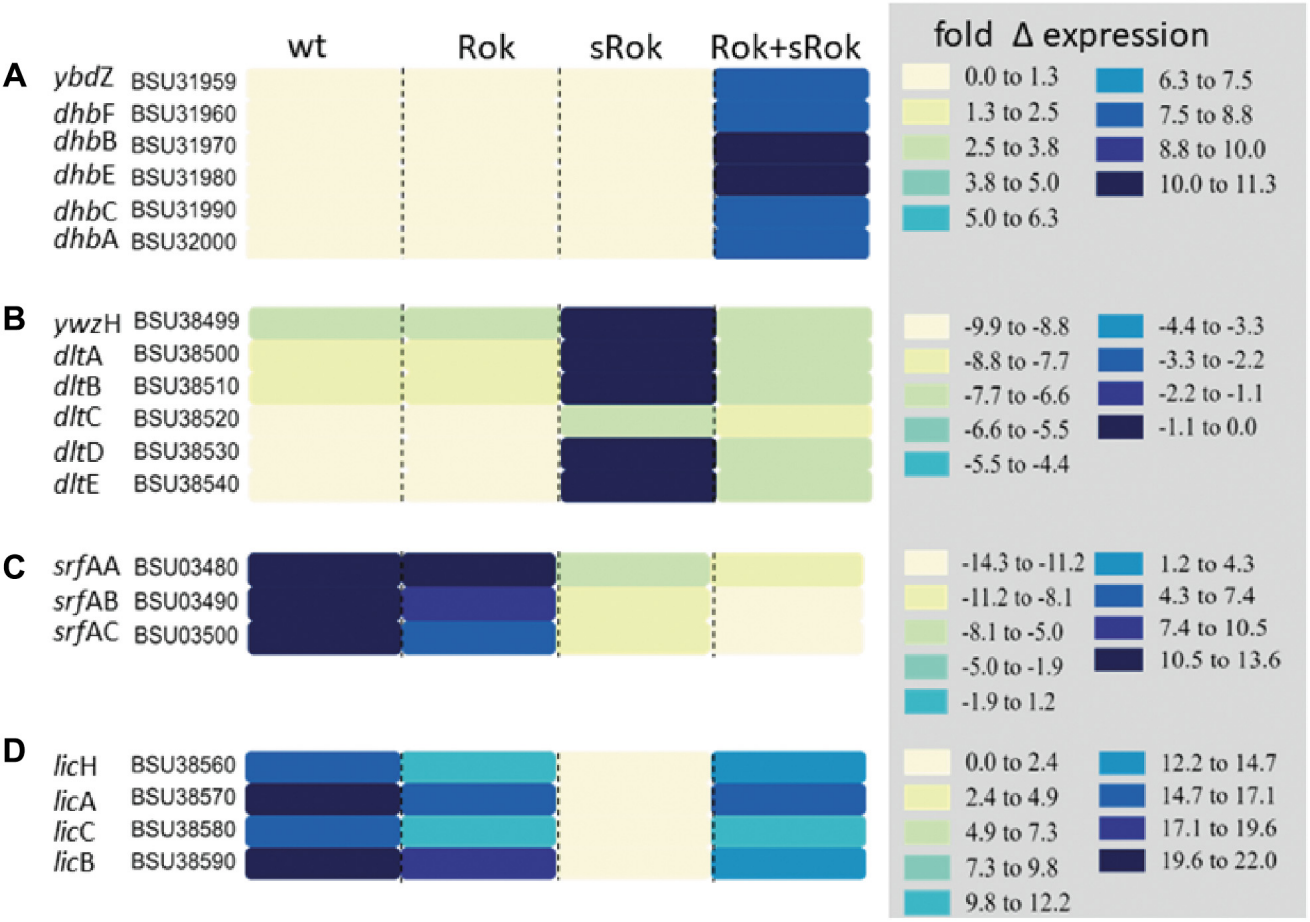
Differential effects of either Rok, sRok and Rok + sRok on sets of functionally related *B. subtilis genes*. Heat map representations of genes involved in the synthesis of bacillibactin (**A**), incorporation of d-alanine in teichoic acids (**B**) or the non-ribosomally synthesized lipopeptide antibiotic surfactin (**C**) and lichenysin D (**D**). Differential expression profiles were generated using the Funage Pro webserver (46). Note that the range of the color scale indicating the expression levels is different for each panel.

The results above show that expression of Rok, sRok and Rok + sRok affect non-identical sets of DEGs. Possibly, the observed differences in DEGs may underlie alterations of distinct pathways or regulons. To study this possibility the differential RNAseq data for strains expressing *rok*, *srok* or *rok* + *srok* with respect to the Δ*rok* strain were uploaded to the Funage Pro server that allows automatic analysis of gene set enrichment (46). Evidence that Rok, sRok and Rok + sRok indeed affect certain operons or pathways differently was obtained. For instance, whereas the presence of Rok or sRok alone did not affect expression of any of the six genes in the *dhb* operon that encodes the biosynthesis of the siderophore bacillibactin (78), all six genes of the operon were highly expressed in the presence of both Rok and sRok (Figure 6A). Other effects were observed for the *dlt* operon that encodes proteins required for incorporation of d-alanine in teichoic acids (79) (Figure 6B). In this case, except for *dlt*C, the presence of sRok hardly affected expression of the *dlt* genes, while Rok caused more than 6-fold decrease in expression of all *dlt* genes. Yet other effects were observed for the genes involved in the non-ribosomally synthesized lipopeptide antibiotics surfactin and lichenysin D (Figure 6C and D). Thus, whereas Rok strongly stimulated expression of both the *srf*A-C and *lic*A-C/H genes, sRok repressed the *srf*A-C genes but hardly affected expression of *lic*A-C/H. Interestingly, the presence of Rok + sRok acted differently on these different operons. As mentioned, whereas Rok and sRok alone did not affect much the bacillibactin genes, these genes were upregulated in the presence of Rok + sRok. Expression of the *dlt* genes in the presence of Rok + sRok was similar to that observed for Rok alone, suggesting that sRok is unable to alter Rok-mediated expression of these genes. The opposite though was observed for the surfactin genes, in which very similar expression profiles were observed for Rok + sRok and sRok alone.

Altogether, the RNAseq data analysis demonstrate that Rok and sRok alter the expression of non-identical sets of genes and that Rok and sRok affect each other′s regulatory activity.

### Presence of Rok and sRok sequences suggest multiple horizontal gene transfer events

**Figure 7.**
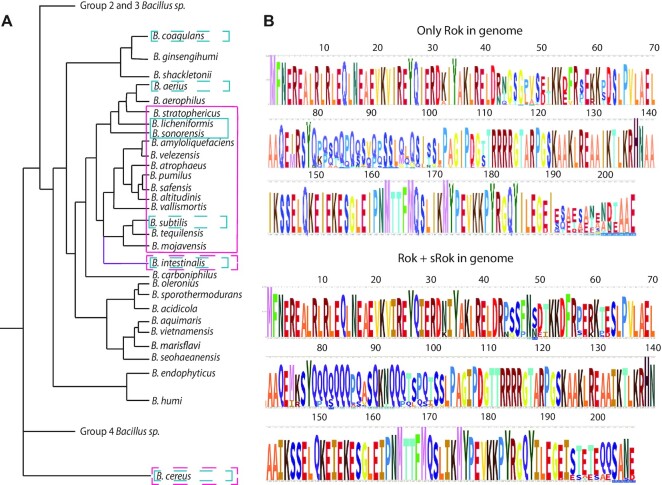
Rok and sRok are only present in a subset of *Bacillus* sp. (**A**) Phylogenetic tree of *Bacillus* sp. Group 1 adapted from Wang and Sun (80) and extended with *B. intestinalis* according to Tetz and Tetz (98). The distances between species are not on scale. A purple line was used for *B. intestinalis* as its exact position is currently unknown. Magenta indicates the presence of a *rok* gene in the genome and blue an *srok* gene. The presence of a gene in only the minority of the genomes available is indicated with dashed lines. (**B**) Sequence logos of the Rok sequences of genomes without (top) or with (bottom) an *srok* gene present.

sRok was first identified on the *B. subtilis* plasmid pLS20 (24). Its presence on a plasmid implies that sRok is not always present in all *Bacillus* species and strains therein. Also, Rok itself was found to be present in only a subset of *Bacillus* species (15). An extensive search in the NCBI protein database extracted 1085 sequences annotated as (s)Rok protein. The *Bacillus* species that contain a *rok* gene form a cluster within group 1 of *Bacillus* species (Figure 7A) (80). While in most species only *rok* was found, some *Bacillus* species have both the *rok* and *srok* gene on their chromosome, most notably *B. licheniformis, B. sonorensis* and *B. subtilis subsp. spizizenii*. Most likely, *srok* was introduced to these lineages via separate horizontal gene transfer events as its location on the chromosome is not conserved in contrast to *rok* (24). A single horizontal gene transfer event was previously proposed for *rok*, where it was introduced in the common ancestor of the *B. subtilis - B. licheniformis–B. amyloliquefaciens* group (15,24). We investigated whether the Rok protein in *Bacillus* species containing also an *srok* gene was adapted to the presence of this binding partner. When comparing the sequence logos of Rok from *Bacillus* species encoding sRok or not, only minor differences in amino acid occurrences were observed, for example at position 75 where both isoleucine and methionine can be found (Figure 7B). Most likely, these minor differences arise from general differences between the *Bacillus* species and the number of Rok sequences used per sequence logo (620 Rok sequences without sRok, 110 sequences with sRok) rather than adaptation of the Rok protein.

## DISCUSSION

**Figure 8.**
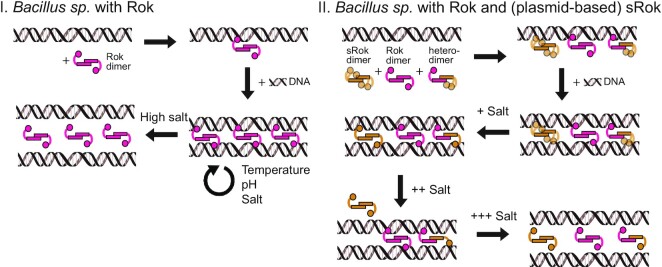
The proposed mechanisms of DNA bridging by (s)Rok. In *Bacillus sp*. with *rok* on their genome (I), Rok binds DNA as a dimer without associating into nucleoprotein filaments. Dimeric Rok acts as bridging unit. Rok dimers cluster cooperatively in between two DNA duplexes, not due to dimer-dimer interactions, but due to high local DNA concentration which drives association and bridging by additional dimers. The DNA bridging activity of Rok is not sensitive to changes in physico-chemical conditions (temperature, pH and salt). When sRok is present, DNA bridging activity can be modulated by salt concentration. In *Bacillus sp*. where both *rok* and *srok* are present either on the genome or on a plasmid (II), a mix of homo- and heterodimers exist. Rok homodimers behave as in (I), while sRok homodimer DNA binding is first stimulated and then inhibited by salt. Heterodimers exhibit average behaviour of the two proteins.

In this work we have discovered that Rok is a DNA bridging protein. This feature is in line with the recent finding that, besides being a transcriptional regulator, Rok plays an important role in chromosome organization by formation of long-ranged loops (23). Formation of such loops is most likely a direct consequence of the DNA bridging capacity of Rok that we established here. Detailed analysis showed that DNA bridging by Rok is only mildly sensitive to changes in physico-chemical conditions (Figure 2). H-NS, MvaT and Lsr2 can use these conditions to switch between DNA bridging and formation of a nucleoprotein filament along DNA, causing DNA stiffening (51–53,81). The bridging activity of H-NS and MvaT can be modulated by both monovalent (Na^+^, K^+^) and divalent (Mg^2+^, Ca^2+^) cations (27,28,53,82). For Lsr2, the effect of changes in ionic strength on the protein's DNA binding properties has not been investigated in detail (51). The formation of either nucleoprotein filaments or DNA-protein-DNA bridges by H-NS family proteins is sensitive to temperature, pH and salt, which facilitates cellular response to environment. We did not observe such a switch for Rok, instead only DNA compaction was observed (Figure 1A), which most likely can be attributed by DNA bridging. It has been reported that binding of Rok's isolated DNA binding domain to DNA causes a bend of around 25° (66). Therefore, it cannot be fully excluded that DNA bending contributes to the DNA compaction behavior of Rok. We propose that Rok bridges DNA by employing dimeric Rok as bridging units (Figure 8). This is different from H-NS-mediated DNA bridging which requires the formation of an H-NS multimer (27). At least 100 bp were needed to recover DNA (Supplementary Figure S2), which indicates that multiple dimers are needed to form a stable Rok–DNA bridge. Therefore, in our model, Rok dimers cluster cooperatively due to the high local DNA concentration adjacent to existing Rok mediated bridges (Figure 8). We cannot rule out the formation of oligomers once Rok is bound to the DNA. Earlier studies suggest that Rok is capable of forming oligomers in solution (66), but we were not able to predict oligomeric structures with Alphafold2. Possibly, oligomers are only formed in solution at very high protein concentrations. Similarly, oligomer formation may be favored while bridging DNA due to the intrinsic cooperative nature of this process. This might be comparable to HU switching from its DNA bending to DNA stiffening mode which involves oligomerization along the DNA at high protein concentrations (83). But, as we did not observe DNA stiffening upon binding of Rok, it remains unclear if Rok forms oligomers when bound to DNA and whether this is needed for stable bridge formation.

Although the sequence similarity of Rok with H-NS, MvaT and Lsr2 is low, these proteins share a similar domain organization. Structural studies have revealed that H-NS, Lsr2 and MvaT have an N-terminal oligomerization domain consisting of two dimerization sites, a C-terminal DNA binding domain and a flexible linker region (9,84–87). Rok has a C-terminal DNA binding domain as well and the N-terminal domain was suggested to be responsible for oligomerization (66). A main difference we identified previously, is the presence of a neutral linker for Rok, while the other proteins have a clear asymmetrical charge distribution (8). Here, we showed that this neutral linker is not responsible for the lack of a strong response of Rok to changes in biologically-relevant conditions as we suggested previously (8). However, we cannot rule out that the subtle changes in Rok's DNA bridging capacity under several physico-chemical conditions might in fact affect gene expression. Removal of the neutral linker decreased the DNA binding cooperativity of Rok and we showed that Rok Δ75–96 can (partially) complement the absence of *hns* in *E. coli*. Therefore, the detrimental effect of ectopic Rok and sRok expression on *E. coli* growth may be due to the high cooperativity in DNA binding of Rok and sRok.

Contrary to Rok, sRok is responsive to different KCl concentrations and is able to form heterodimers with Rok, making the Rok:sRok complex also osmo-sensitive (Figure 4). In *Bacillus sp*. where both Rok and sRok are present, homo- and heterodimers exist in an unknown ratio, making part of the complexes osmo-sensitive (Figure 8). Therefore, we propose that protein-protein interactions with Rok are a more important, primary mechanism to regulate genes repressed by Rok than changes in environmental conditions. Because *srok* is only present in a subset of *Bacillus sp*., either on a plasmid or encoded on the chromosome (Figure 7), this hints at the existence of other convergently evolved sRok-like NAPs in sRok deficient *Bacillus sp*. Also the fact that *srok* is nearly exclusively present together with *rok*—in contrast to *rok* itself, which is frequently found alone—suggests that sRok is rather a modulator of Rok activity than a main NAP itself.

Our RNAseq data and Funage Pro analysis show that both Rok and sRok on their own and their combination have a unique, only partially overlapping, regulon. Particularly the observation that the combination of Rok and sRok is not merely adding the individual genes/regulons together, suggests an extensive interplay between the two proteins. However, we cannot exclude indirect effects on transcription mediated by the effect of Rok and sRok on the expression level of other transcriptional regulators and/or proteins involved in chromatin organization (Supplementary Figure S10A). HBsu (generally referred to as HU) was downregulated upon ectopic expression of (s)Rok, while its level of transcription is enhanced in the wildtype compared to the *Δrok* strain. Second, the combined expression of Rok and sRok activated gyrase and topoisomerase genes. Which parts of the different expression patterns observed in Figure 5 are direct consequences of (s)Rok and which are indirect effects, remains therefore unanswered.

The distinct regulons might also reflect the different osmo-sensitivity of the two proteins and their combined complex (Figure 4). The known regulon of Rok so far mainly contains genes with a function in membrane maintenance and antimicrobial activity (15,17,88,89). This suggests that the Rok regulon is not directly involved in the response to environmental cues, in contrast to for example the *proU* operon in *E. coli*, which is regulated by H-NS (61,90). This might explain why Rok has not evolved a strong response to changes in physico-chemical conditions. We compared the previously determined Rok regulon (19) with the genes that change significantly upon salt shock (91) and indeed very few genes overlap (Supplementary Figure S10B). Also the effects on transcription of these genes are either very minor or do not significantly change between the overexpression of Rok, sRok or Rok + sRok.

The stronger responsiveness of sRok to salt *in vitro* suggested that sRok regulates genes with a function in salt response and the formation of a complex between Rok and sRok, binding to (environmentally sensitive) operons not directly bound by Rok (or sRok) might be observed. It is a possibility that this happens at other osmo-sensitive genes or that indirect effects as described above might play a role. It makes it tempting to speculate that the habitat of the specific *Bacillus* species having both a *rok* and *srok* gene is different from that of other *Bacillus sp* containing only *rok*. It has been found that *B. licheniformis* and *B. sonorensis* have a slightly higher salt tolerance than *B. subtilis* (92), which might explain the need for a NAP (sRok) that is stimulated by higher salt concentrations.

It has been noted previously that two strains with both Rok and sRok on their chromosome showed a lack of competence development (24,93). This might be due to *comK* repression by both Rok and sRok, however this was not visible in our data due to different growth conditions which do not induce *comK* expression. Further research into sRok's function *in vivo* is needed to see to what extent the direct regulon of sRok is different from that of Rok and what differences might be due to indirect effects via other regulators of transcription. Also, whether sRok is sufficiently transcribed to play a significant role in the respective *Bacillus* strains remains an open question.

Another transcriptional regulatory partner of Rok is DnaA. These two proteins cooperate in transcriptional repression of various genes (94), but it was shown that DnaA is not needed for chromosomal loop formation by Rok (23). Although it is unknown if DnaA affects the ability of Rok to bridge DNA, these observations strengthen the hypothesis that interaction with other proteins is one of the main ways to modulate Rok-mediated gene regulation. Future research is needed to determine if besides DnaA and sRok other proteins can influence the role of Rok in regulation. An example of a Rok antagonist is ComK that can relieve gene repression mediated by Rok at the *comK* promoter (95). There is no direct evidence that this involves physical interaction between the two proteins, but binding to DNA is not mutually exclusive. Activation of *comK* transcription is believed to be induced by alteration of local nucleoprotein structure, permitting transcription initiation (95). Based on the robustness of Rok in binding to DNA we expect the existence of similar antagonistic factors operating at other sites across the chromosome. This bears similarity to regulation of transcription through local chromatin remodeling at complex promoters in *E. coli* (96). Studying the protein partners of Rok might shed light on how the modulation of Rok works in *B. subtilis* and provide new insights into gene regulation by NAPs in general.

## DATA AVAILABILITY

The datasets generated during the current study are available from the 4TU repository (https://data.4tu.nl) with DOI 10.4121/18134129. The RNA-seq data has been deposited as BioProject at the NIH, National Library of Medicine, National Center for Biotechnology Informationn under ID: 881227.

## Supplementary Material

gkac1064_Supplemental_Files
