# Supplementary material for: The B. subtilis Rok protein is an atypical H-NS-like protein irresponsive to physico-chemical cues
Source: Nucleic Acids Res. 2022 Nov 21;50(21):12166–85. doi: 10.1093/nar/gkac1064 (PMC9757077; doi:10.1093/nar/gkac1064)
Supplement: gkac1064_Supplemental_Files [file gkac1064_supplemental_files.zip › Captions table S6, S7 and S8.docx]

**Table S6. Differentially expressed genes upon sRok expression** The locus and coordinates are given as on *Bacillus subtilis subsp. subtilis str. 168* reference genome (GenBank identifier AL009126.3). The differences were calculated with respect to the *Δrok* strain.

**Table S7. Differentially expressed genes upon Rok expression** The locus and coordinates are given as on *Bacillus subtilis subsp. subtilis str. 168* reference genome (GenBank identifier AL009126.3). The differences were calculated with respect to the *Δrok* strain.

**Table S8. Differentially expressed genes upon Rok+sRok expression** The locus and coordinates are given as on *Bacillus subtilis subsp. subtilis str. 168* reference genome (GenBank identifier AL009126.3). The differences were calculated with respect to the *Δrok* strain.
