# Supplementary material for: The B. subtilis Rok protein is an atypical H-NS-like protein irresponsive to physico-chemical cues
Source: Nucleic Acids Res. 2022 Nov 21;50(21):12166–85. doi: 10.1093/nar/gkac1064 (PMC9757077; doi:10.1093/nar/gkac1064)
Supplement: gkac1064_Supplemental_Files [file gkac1064_supplemental_files.zip › Supplementary figures V2.pdf]

Supplementary figures

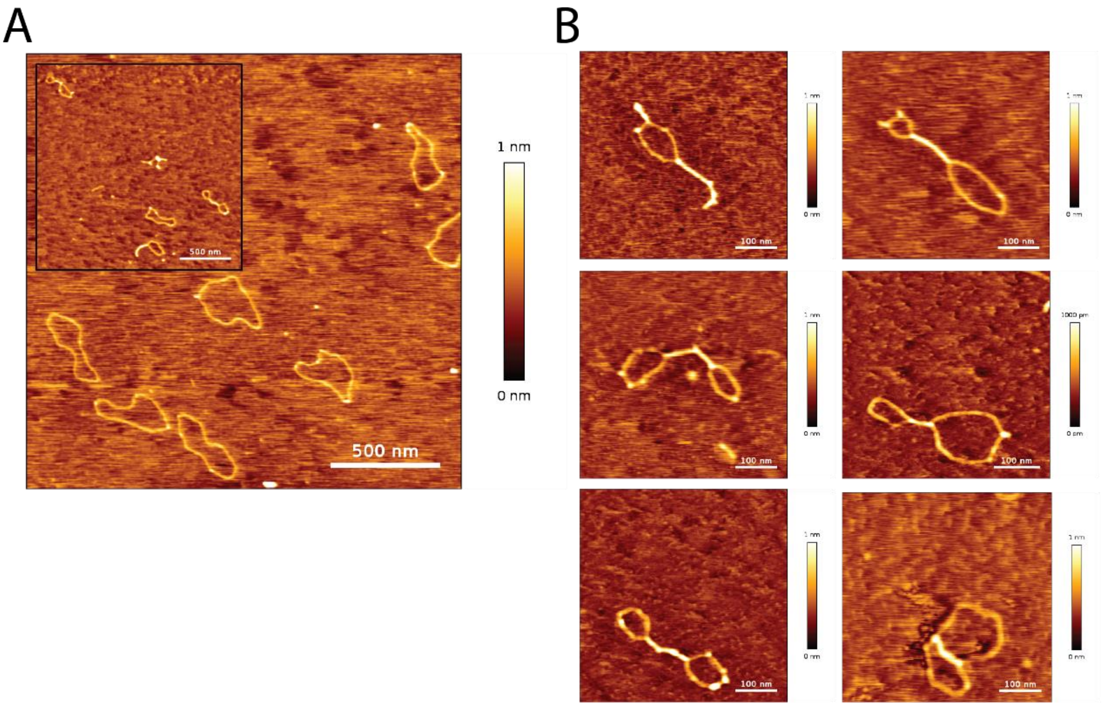

Figure S1. Rok bridges DNA as observed by AFM imaging A) Nicked pUC19 molecules incubated without Rok or with Rok at a concentration of 200 nM (insert). B) Close-ups of representative Rok-DNA complexes.

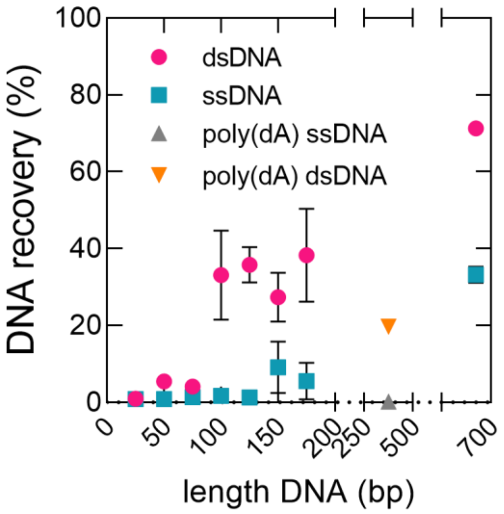

Figure S2. Rok bridges dsDNA but not ssDNA DNA recovery (%) as a function of the length of prey DNA in bp in the presence of 0.27 µM Rok with 50 mM KCl at 25°C. The bait DNA was the 685 bp dsDNA as used for the other bridging experiments. Data are plotted as mean values and the error bars represent the standard deviation from three independent measurements.

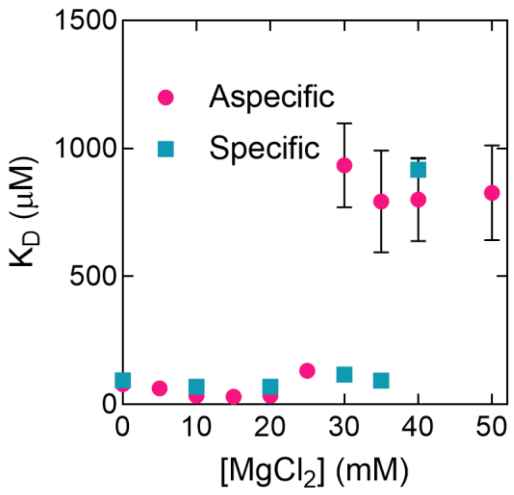

Figure S3. $MgCl_2$ does not affect the DNA binding affinity of Rok Kd-values (µM) obtained from fitting protein titration data to the McGhee-von Hippel equation as a function of $MgCl_2$ concentration. The DNA used was 78 bp in length with or without a specific Rok binding site and the Rok concentration was varied between 0.125 and 16 µM. The final measurement buffer consisted of 10 mM Tris HCl pH 8, 150 mM KCl, 5% glycerol, 0.05% Tween20 and 0.08 mg/ml acetylated BSA. $MgCl_2$ was added accordingly. Each data point was measured at least in triplicate and error bars represent the standard deviation. Some error bars are hidden behind the data points due to their small size.

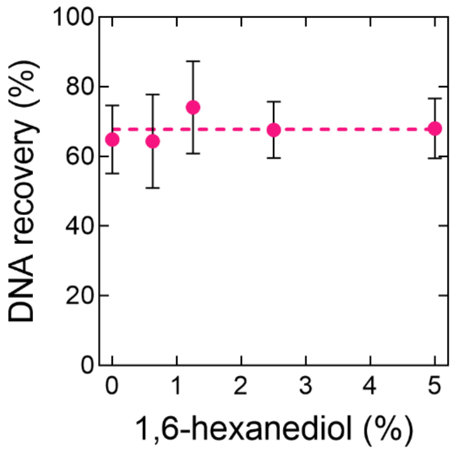

Figure S4. 1,6-hexanediol cannot prevent Rok-DNA bridges being formed DNA recovery (%) as a function of added 1,6-hexanediol (%) in the presence of 0.27 µM Rok with 50 mM KCl at 25°C. Data are plotted as mean values and the error bars represent the standard deviation from three independent measurements. Dashed line serves as line to guide the eye.

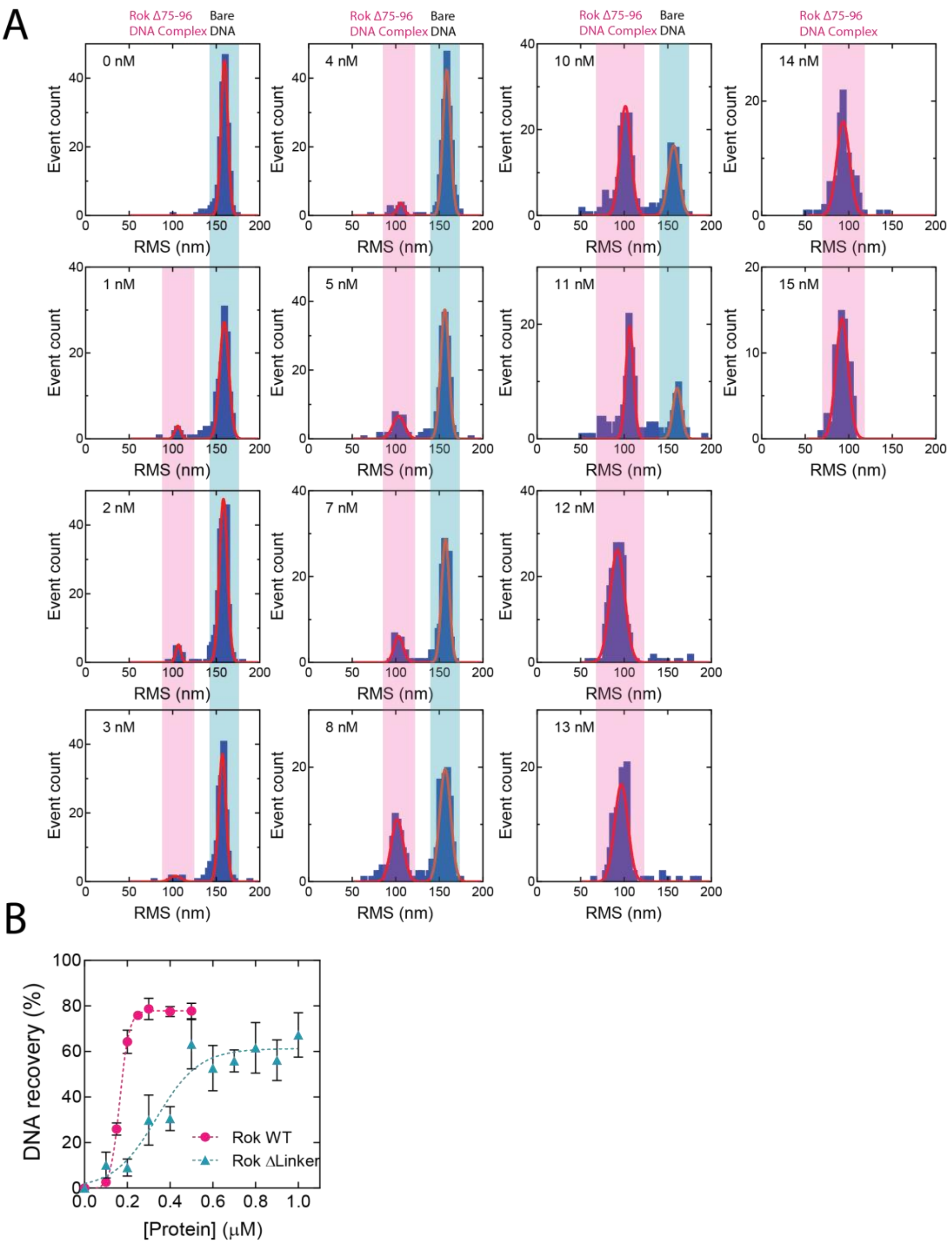

Figure S5. Rok Δ75-96 compacts and bridges DNA but with decreased cooperativity. A) Histograms of Root Mean Square displacement (RMS) obtained for 32%GC DNA as a function of Rok Δ75-96 at concentrations of 0, 1, 2, 3, 4, 5, 7, 8, 9, 10, 11, 12, 13, 14 and 15 nM as measured by TPM in the presence of 50 mM KCl. The histograms were fitted to Gaussian distributions, in which the RMS value at ~150 nm represents bare DNA and the population with an RMS at ~100 nm represents DNA bound by Rok Δ75-96. The bare DNA and Rok-DNA complex populations are highlighted with a light blue and magenta box, respectively. The data for each concentration originates from at least two independent measurements. B) DNA recovery (%) as a function of protein concentration in μM in the presence of 50 mM KCl at 25°C. For reference, Rok WT is shown (reproduced from figure 2A). Data

are plotted as mean values and the error bars represent the standard deviation from three independent measurements. Dashed lines serve as lines to guide the eye.

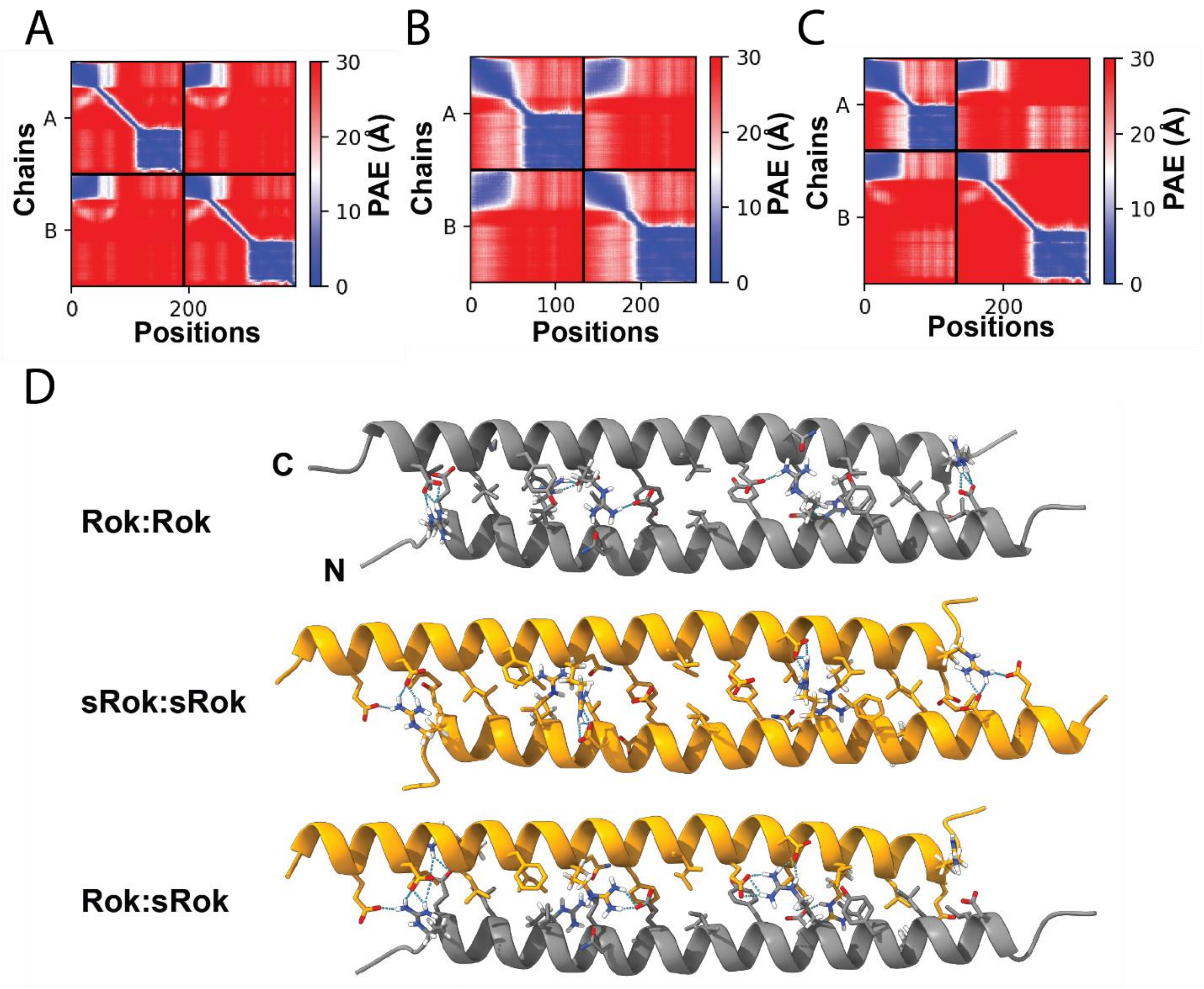

Figure S6. Alphafold2 predicts (s)Rok homo- and heterodimers with high confidence A) Predicted aligned error (PAE) plot of the Rok homodimer, B) sRok homodimer and C) Rok:sRok heterodimer structure. D) The dimerization domains of homodimers Rok (grey, amino acids 1 to 46) and sRok (orange, amino acids 1 to 48) and heterodimer Rok:sRok as predicted by AlphaFold2. Amino acids that are located within the coiled-coil dimerization interface are drawn as sticks.

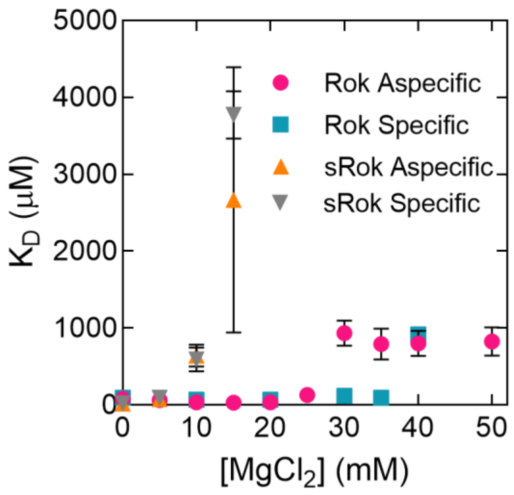

Figure S7. $MgCl_2$ does affect the DNA binding affinity of sRok Kd-values (µM) obtained from fitting protein titration data to the McGhee-von Hippel equation as a function of the $MgCl_2$ concentration. The DNA used was 78 bp in length with or without a specific Rok binding site and the sRok concentration was varied between 0.125 and 16 µM. The Rok data was reproduced from figure S2 for comparison. The final measurement buffer consisted of 10 mM Tris HCl pH 8, 150 mM KCl, 5% glycerol, 0.05% Tween20 and 0.08 mg/ml acetylated BSA. $MgCl_2$ was added accordingly. Each data point was measured at least in triplicate and error bars represent the standard deviation. Some error bars are hidden behind the data points due to their small size.

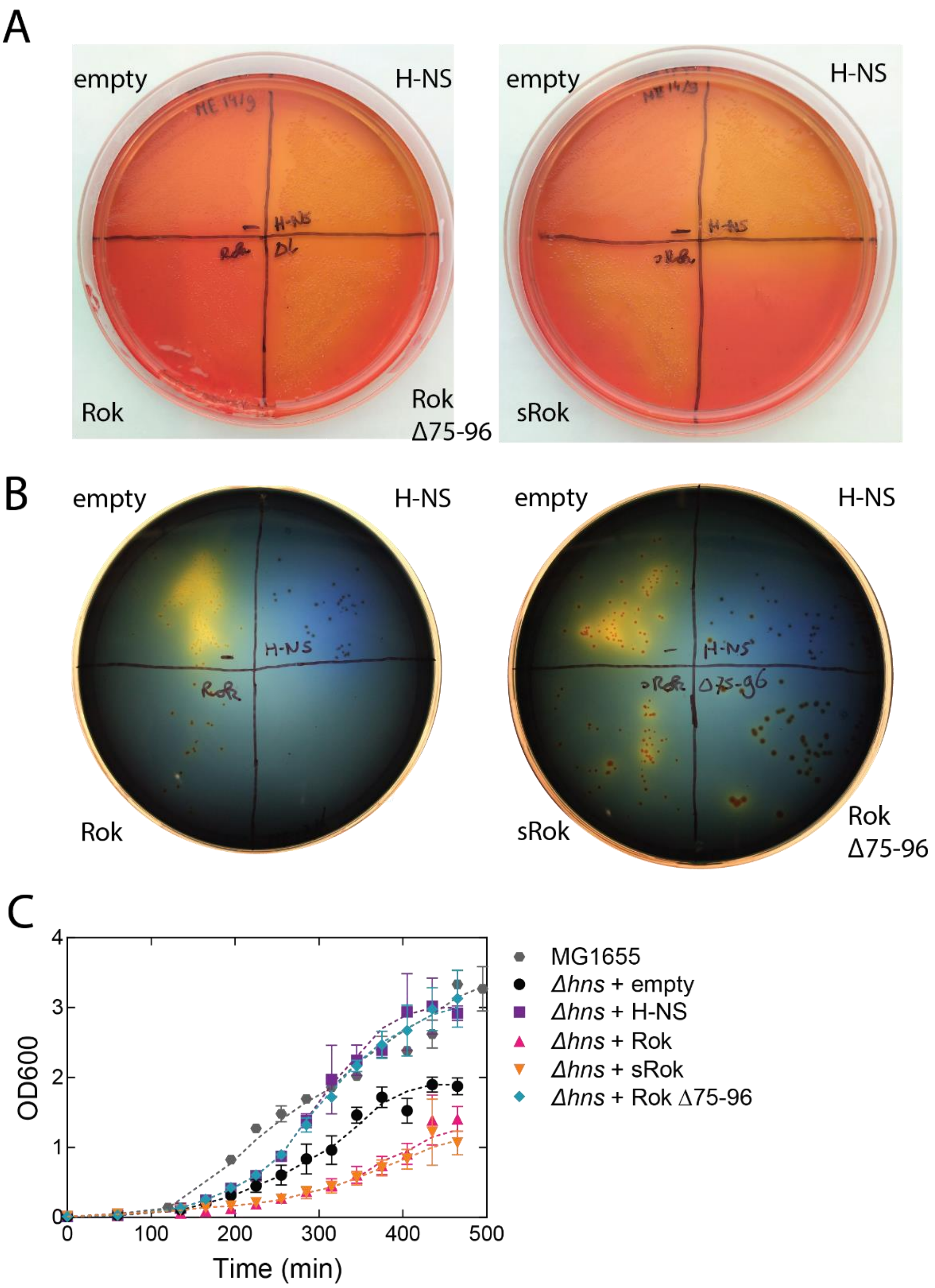

Figure S8. Rok and sRok cannot complement a *Δhns* phenotype, but Rok Δ75-96 can A) Complementation of *bgl* operon repression tested on MacConkey agar plates supplemented with 0.4% salicin. *E. coli* NT135 cells were transformed with either an empty pUC19 plasmid or with an insert containing the *hns* promoter followed by the *hns, rok, srok* or *rokΔ75-96* coding sequence. B) Complementation of *bgl* operon repression tested on BTB indicator plates supplemented with 0.5% salicin, using the same cells as in A. C) Growth curves in LB medium over time. Data are plotted as mean values and the error bars represent the standard deviation of three independent growth curves. Dashed lines serve as lines to guide the eye.

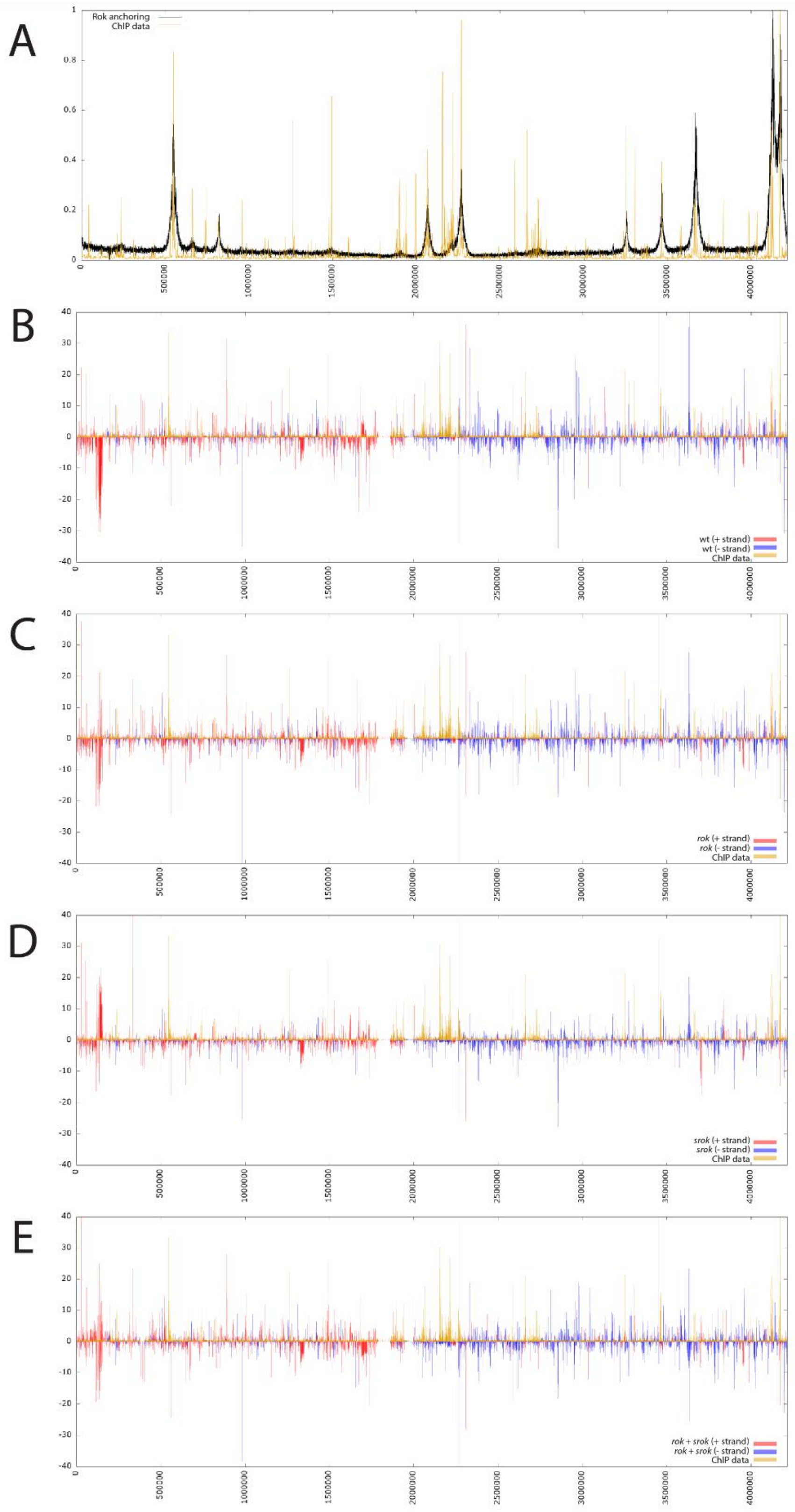

Figure S9. Rok, sRok and Rok+sRok affect multiple loci across the *B. subtilis* genome. A) Overlay of previously published ChIP data (1) and DNA coverage data (2) for Rok. B-E) Overlay of the expression levels on the + strand, - strand for wt (B), Rok (C), sRok (D) and Rok+sRok (E). For reference, the ChIP data for Rok was included (1).

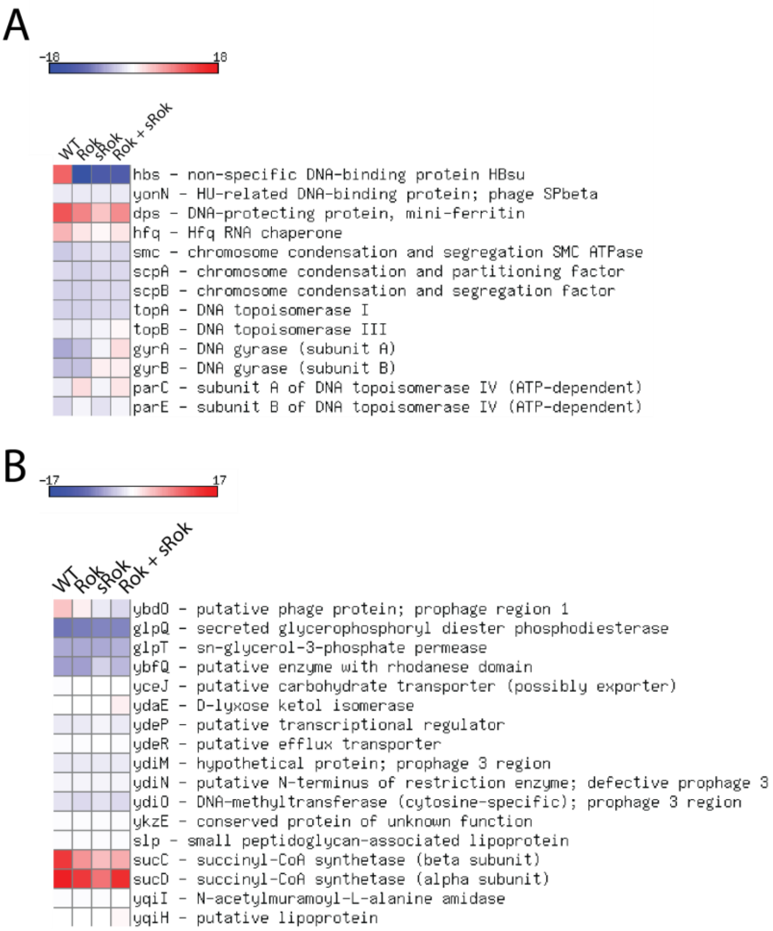

Figure S10. Effect of Rok, sRok and Rok+sRok on osmo-regulated genes and proteins involved in chromatin organization. Heat map representations of a set of proteins involved in chromatin organization (A) or osmo-regulated genes (B) in response to *rok*, *srok* and *rok+srok* expressed from an ectopic promoter.

Table S1. Oligonucleotides used for cloning and mutagenesis. The name and sequence of the primer are given and the plasmid(s) in which construction they were used are indicated.

Name primer	Resulting plasmid	Sequence (5'-3')
Rok_fragment_F	pRD231/pRD415	GAAATAATTTTGTTTAACTTTAAGAAGGAGATATA CATATGTTTAATGAAAGAGAAGCTTTGCGC
Rok_fragment_R	pRD231/pRD415	CCTTTCGGGCTTTGTTAGCAGTTATTCGTTTGCT GATTCTGCAGATTCGA
Rok_vector_F	pRD231/pRD415	GCGCAAAGCTTCTCTTTCATTAAACATATGTATAT CTCCTTCTTAAAGTTAAACAAAATTATTTC
Rok_vector_R	pRD231/pRD415	TCGAATCTGCAGAATCAGCAAACGAATAACTGCT AACAAAGCCCGAAAGG
Rok_delta_linker F	pRD415	CGGCTCAGGAAATCAGAGCCGGTATACCAGACG G
Rok_delta_linker R	pRD415	CCGTCTGGTATACCGGCTCTGATTTCCTGAGCC G
sRok_pET30b F	pRD411	AACTTTAAGAAGGAGATATACATATGCTGACCGA ACGTCAGGC

sRok_pET30b R	pRD411	CCTTTCGGGCTTTGTTAGCAGTTACATTTCTTCAAAGCGAT
sRok_pET30b_vector F	pRD411	ATCGCTTTGAAGAAATGTAACTGCTAACAAAGCCCGAAAGG
sRok_pET30b_vector R	pRD411	GCCTGACGTTCGGTCAGCATATGTATATCTCCTTCTTAAAGTT
ivc_H-NS_pUC19_F	pRD408	GAGCTCGGTACCCGGGGATCTTCTGGCTAATTTTATGAAA
ivc_H-NS_pUC19_R	pRD408	CCTGCAGGTCGACTCTAGAGCAAGTGCAATCTACAAAAGA
ivc_pUC19_H-NS_F	pRD408	TTTCATAAAATTAGCCAGAAGATCCCCGGGTACCGAGCTC
ivc_pUC19_H-NS_R	pRD408	TCTTTTGTAGATTGCACTTGCTCTAGAGTCGACCTGCAGG
sRok_pUC19 F	pRD410	GATAGGGGGTACATTGAGGAATGCTGACCGAACGTCAGGC
sRok_pUC19 R	pRD410	CAAGCAGTTTTTCTTTATATTTACATTTCTTCAAAGCGAT
sRok_pUC19_vector F	pRD410	ATCGCTTTGAAGAAATGTAAATATAAAGAAAAACTGCTTG
sRok_pUC19_vector R	pRD410	GCCTGACGTTCGGTCAGCATTCCTCAATGTACCCCCTATC
Rok_pH-NS F	pRD424/pRD412	ATAAGTTTGAGATTACTACAATGTTTAATGAAAGAGAAGC
Rok_pH-NS R	pRD424/pRD412	CAAGTGCAATCTACAAAAGATTATTCGTTTGCTGATTCTG
pH-NS_Rok F	pRD424/pRD412	GCTTCTCTTTCATTAAACATTGTAGTAATCTCAAACTTAT
pH-NS_Rok R	pRD424/pRD412	CAGAATCAGCAAACGAATAATCTTTTGTAGATTGCACTTG
Forward pET30b	pRD461	CCGCTATCGCTACGTGACTGGGTCATGGCTGCGCCCCGACACCCG
Reverse pET30b	pRD461	AGCCATGACCCAGTCACGTAGCGATAGCGGAGTGTATACTGGCTT
Rok N-term F	pRD461	CATCATCATCATCATCATtaaCTGCTAACAAAGCCCGAAAG
Rok 46-191 his R	pRD461	TTAATGATGATGATGATGATGTTCGTTTGCTGATTCTGCAGATTCGA
oAND314	pAND520	TTTTaagcttAAAGGAGAGATATAAATGCTTACAGAAAGAC
oAND315	pAND520	TTTTgtcgacGTGTTACATTTCTTCGAATCTATAATAACC
oAND316	pAND521/pAND522	TTTTgtcgacAAAGGAGAGATATAAATGTTTAATGAAAGAG
oAND317	pAND521/pAND522	TTTTgctagcGAAAAAGAAAACAAACCTTCACAGAAAAAACCTC

Table S2. Plasmids created for this study. For each plasmid, the backbone, insert, antibiotics resistance are given. All plasmids are deposited to Addgene and the respective identification numbers are given.

**Name**	**Backbone**	**Insert**	**Resistance**	**Addgene number**
pRD231	pET30b	Rok	Kanamycin	178195
pRD411	pET30b	sRok	Kanamycin	178196
pRD415	pET30b	Rok Δ75-96	Kanamycin	178197
pRD461	pET30b	Rok 6xhis	Kanamycin	178198
pRD408	pUC19	p*hns* + H-NS	Ampicillin	178199
pRD410	pUC19	p*hns* + sRok	Ampicillin	178200
pRD412	pUC19	p*hns* + Rok Δ75-96	Ampicillin	178201
pRD424	pUC19	p*hns* + Rok	Ampicillin	178202

Table S3. Oligonucleotides used for single-stranded DNA substrates. Highlighted sequences were found to be favorable for Rok binding by Duan et al. 2018 (3).

**Length**	**Sequence**
25 bp	GGAGTAGTATGGTAATAACTATTTT
50 bp	GCAAATATATAATGTATAAGTTCTGGAGTAGTATGGTAATAACTATTTTA
75 bp	CTTAGTGGCAAATATATAATGTATAAGTTCTGGAGTAGTATGGTAATAACTATTTTAT TTTCGATAGCTTGAATG
100 bp	GATATATGGACTCTTAGTGGCAAATATATAATGTATAAGTTCTGGAGTAGTATGGTA ATAACTATTTTATTTTCGATAGCTTGAATGTTTATTTTCCCAG
125 bp	ATTTAGTCTATTCGATATATGGACTCTTAGTGGCAAATATATAATGTATAAGTTCTGG AGTAGTATGGTAATAACTATTTTATTTTCGATAGCTTGAATGTTTATTTTCCCAGAGA CATTAGGTG
150 bp	TTTCTCCAATTGATTTAGTCTATTCGATATATGGACTCTTAGTGGCAAATATATAATG TATAAGTTCTGGAGTAGTATGGTAATAACTATTTTATTTTCGATAGCTTGAATGTTTAT TTTCCCAGAGACATTAGGTGGTCTTTCAAACTC
175 bp	TCTCTTCAAATGTTTTCTCCAATTGATTTAGTCTATTCGATATATGGACTCTTAGTGG CAAATATATAATGTATAAGTTCTGGAGTAGTATGGTAATAACTATTTTATTTTCGATA GCTTGAATGTTTATTTTCCCAGAGACATTAGGTGGTCTTTCAAACTCAATAATGTGG GT

Table S4. Oligonucleotides used for MST DNA substrates. Highlighted sequence was found to be favorable for Rok binding by Duan et al. 2018 (3).

Aspecific top strand	Cy5-CGGCGCAAATTCGTGACCAGTTGCATCAGCTGCGTGAGCTGTTTATCGC AGCATCGTAACAGGATAGTGAAGAAGACT
Aspecific bottom strand	AGTCTTCTTCACTATCCTGTTACGATGCTGCGATAAACAGCTCACGCAGCTGA TGCAACTGGTCACGAATTTGCGCCG
Specific top strand	Cy5-CGGCGCAAATTCGTGACCAGTTGCATCAGCTACTAGAGCTGTTTATCGC AGCATCGTAACAGGATAGTGAAGAAGACT

Specific bottom strand	AGTCTTCTTCACTATCCTGTTACGATGCTGCGATAAACAGCTCTAGTAGCTGATGCAACTGGTCACGAATTTGCGCCG

Table S5. Effects of ectopic expression of Rok as fold change of selected set of genes reported before to be regulated by Rok. The differences in expression between each of the strains and the Δ*rok* strain are calculated by taking the square root of the difference between the normalized mean counts of each of the strains with respect to those of the Δ*rok* strain, for each of the genes.

operon	Gene(s)	Reference	reported effect Rok	These studies (differential expression with respect to Δ*rok* strain)			
				wt strain	ectopic expression		
					Rok	sRok	sRok+Rok
	*htp*X	(4)	Min	-5.38	-6.05	-5.20	4.43
	*com*K	(5)	Min	-1.24	-1.92	-1.82	-1.92
	*sdp*A		Min	1.29	-1.05	-0.92	-1.05
sdp	*sdp*B		Min	1.27	-0.90	-0.72	-0.91
	*sdp*C	(6)	min	4.61	-3.23	-1.38	-3.39
	sboA		Min	2.76	-10.51	13.36	-10.13
	sboX		Min	-2.06	-4.96	6.28	-4.89
	albA		Min	-6.41	-7.29	12.44	-7.16
	albB		Min	-6.32	-7.56	11.28	-7.27
alb	*albC*		Min	-5.01	-5.72	8.78	-5.53
	albD		Min	-2.24	-2.56	2.83	-2.55
	albE		Min	-2.94	-3.31	4.55	-3.27
	albF		Min	-2.66	-2.98	4.02	-2.92
	albG	(6)	Min	-1.97	-2.37	-3.05	-2.29
	rok	(5)	Min	5.33	7.5	-0.18	11.85
	yybN		Min	-14.70	-19.36	-14.76	-20.37
	yybM		Min	-2.96	-3.91	-3.22	-4.03
ybb	*yybL*		Min	-2.52	-2.92	-2.64	-2.94
	yybK		Min	-2.93	-3.23	-2.84	-3.26
	yybJ	(2, 6)	Min	-4.08	-4.78	-3.70	-4.85
	sunA		Min	-34.10	-82.33	-24.08	-82.88
	sunT		Min	-7.43	-8.45	-7.16	-8.47
sun	*bdbA*		Min	-6.12	-8.16	-6.58	-8.18
	sunS		Min	-5.35	-8.39	-6.53	-8.43
	bdbB	(6, 7)	Min	-5.36	-9.24	-6.98	-9.27
	epeX		Min	2.03	1.10	-0.77	1.07
	epeE		Min	0.13	-0.6	-0.57	-0.62
epe	*epeP*		Min	-0.37	-1.00	-0.75	-1.05
	epeA		Min	-2.01	-2.92	-1.91	-3.00
	epeB	(6)	Min	-2.27	-3.23	-2.13	-3.31
	yxaJ	(6)	Min	-4.79	-5.25	-3.63	-5.29
	yxaL	(6)	Min	-3.96	-9.34	7.38	-10.46
	yjcN	(6)	Min	-5.20	-6.54	-5.13	-6.73
	yuaB (=*bsl*A) (indirect)	(8)	min	1.76	4.31	2.98	7.41

1. Smits,W.K. and Grossman,A.D. (2010) The transcriptional regulator Rok binds A+T-rich DNA and is involved in repression of a mobile genetic element in Bacillus subtilis. *PLoS Genet.*, **6**, 1001207.

2. Dugar,G., Hofmann,A., Heermann,D.W. and Hamoen,L.W. (2022) A chromosomal loop anchor mediates bacterial genome organization. *Nat. Genet. 2022 542*, **54**, 194–201.

3. Duan,B., Ding,P., Hughes,T.R., Navarre,W.W., Liu,J. and Xia,B. (2018) How bacterial xenogeneic silencer rok distinguishes foreign from self DNA in its resident genome. *Nucleic Acids Res.*, **46**, 10514–10529.

4. Marciniak,B.C., Trip,H., Fusetti,F. and Kuipers,O.P. (2012) Regulation of ykrL (htpX) by Rok and YkrK, a novel type of regulator in Bacillus subtilis. *J. Bacteriol.*, **194**, 2837–2845.

5. Hoa,T.T., Tortosa,P., Albano,M. and Dubnau,D. (2002) Rok (YkuW) regulates genetic competence in Bacillus subtilis by directly repressing comK. *Mol. Microbiol.*, **43**, 15–26.

6. Albano,M., Smits,W.K., Ho,L.T.Y., Kraigher,B., Mandic-Mulec,I., Kuipers,O.P. and Dubnau,D. (2005) The Rok protein of Bacillus subtilis represses genes for cell surface and extracellular functions. *J. Bacteriol.*, **187**, 2010–9.

7. Denham,E.L., Piersma,S., Rinket,M., Reilman,E., de Goffau,M.C. and van Dijl,J.M. (2019) Differential expression of a prophage-encoded glycocin and its immunity protein suggests a mutualistic strategy of a phage and its host. *Sci. Rep.*, **9**, 2845.

8. Kovács,Á.T. and Kuipers,O.P. (2011) Rok regulates yuaB expression during architecturally complex colony development of Bacillus subtilis 168. *J. Bacteriol.*, **193**, 998–1002.
